# A novel nail polish/graphite/nickel nanoparticles-based disposable screen-printed sensor for enhanced voltammetric detection of mefenamic acid in environmental water samples

**DOI:** 10.1007/s00604-026-08105-4

**Published:** 2026-06-04

**Authors:** Luís Eduardo da Conceição Teixeira, Francisco Walison Lima Silva, Anne Alves Macedo, Octávio P. L. de Souza, João H. A. Ferreira, Thiago C. Canevari, Ricardo Erthal Santelli, Lucas Vinicius de Faria, Fernando Henrique Cincotto

**Affiliations:** 1 Departamento de Química Analítica, Instituto de Química, Universidade Federal do Rio de Janeiro, Rio de Janeiro, RJ 21941-909 Brazil; 2 Multifunctional nanomaterials hybrid laboratory (LABNAHM), Engineering School, Mackenzie Presbyterian University, São Paulo, SP 01302-907 Brazil; 3 National Institute of Science and Technology of Bioanalytics Lauro Kubota (INCTBio-LK), Campinas, SP 13083-970 Brazil

**Keywords:** Mefenamic acid, Electrochemical sensor; Graphite screen-printed carbon electrode; Differential pulse voltammetry, Low-cost and disposable sensor, Environmental samples

## Abstract

A low-cost electrochemical sensor based on a graphite screen-printed carbon electrode modified with nickel nanoparticles was developed for the sensitive determination of mefenamic acid (MFA), an emerging environmental contaminant due to its persistence and incomplete removal in wastewater treatment, which may lead to endocrine-disrupting effects in aquatic systems. The incorporation of nickel nanoparticles into the graphite-based ink significantly increased the electroactive surface area and enhanced electron transfer kinetics, resulting in improved analytical performance. Morphological and structural analyses confirmed the successful integration of nickel nanoparticles into the electrode matrix. Electrochemical characterization using cyclic voltammetry and electrochemical impedance spectroscopy demonstrated higher faradaic currents and lower charge transfer resistance compared to the unmodified electrode. Under optimized conditions, differential pulse voltammetry provided a well-defined oxidation signal for mefenamic acid, with a linear range from 0.5 to 12.20 µmol L⁻¹, a limit of detection of 69.12 nmol L⁻¹, and a sensitivity of 0.434 µA L µmol^− 1^. The sensor exhibited good repeatability (RSD = 3.85%), satisfactory long-term stability (88% signal retention after 30 days), and excellent recovery (96–99%) in tap and river water samples. The proposed platform combines sensitivity, reliability, low cost, and operational simplicity, demonstrating strong potential for on-site environmental monitoring of mefenamic acid.

**Graphical abstract:**

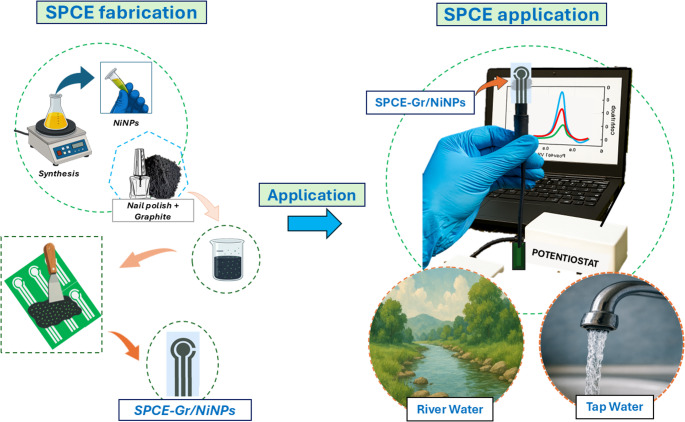

**Supplementary Information:**

The online version contains supplementary material available at 10.1007/s00604-026-08105-4.

## Introduction

Mefenamic acid (MFA), also known as 2-(2,3-dimethylphenyl) amino benzoic acid, is a drug that belongs to the class of nonsteroidal anti-inflammatories (NSAIDs). It is mainly recommended for patients with musculoskeletal and joint disorders, such as osteoarthritis and rheumatoid arthritis, and for the treatment of pain associated with the menstrual cycle [1]. The ingestion of MFA at elevated doses can cause hepatic necrosis, liver damage, vomiting, diarrhea, and malaise, and may even lead to death [2].

Contamination by MFA and other NSAIDs has become an emerging significant environmental concern in many countries due to their widespread production and consumption. These residues can contaminate water bodies and owing to the chemical structure of MFA and the limited efficiency of conventional wastewater treatment processes, aquatic organisms may be exposed to its endocrine-disrupting effects [3]. MFA can affect the aromatase enzyme, potentially alter the production of sex hormones, and impact reproduction [4]. Therefore, the detection of MFA in environmental and biological samples is crucial for the effective monitoring and treatment of wastewater discharged into aquatic systems worldwide.

In the literature, various methods have been employed for the detection of MFA, including electrophoresis [5], high-performance liquid chromatography (HPLC) [6], spectrophotometry [3], and fluorimetry [4]. Although widely used, these techniques present some limitations, such as being time-consuming, costly, requiring complex sample preparation, and involving rigorous analytical protocols. Alternatively, electrochemical methods offer some advantages for MFA sensing, including high sensitivity, rapid response, and minimal sample preparation. For this purpose, a variety of materials have been reported for the modification of glassy carbon (GCE) and carbon paste (CPE) electrodes, including multiwalled carbon nanotubes, lanthanum hydroxide, iron-based complexes, and ruthenium-doped TiO₂ [7–10]. In addition to conventional substrates, electrochemical platforms, such as screen-printed electrodes (SPE), can be applied for the detection of various analytes due to their simplicity, portability, and low cost per analysis [11–14]. Because of this, recently, SPE devices have attracted considerable attention from the scientific community, particularly using modified surfaces [15]. In this scenario, lab-fabricated screen-printed carbon electrodes (SPCE) based on graphite (Gr) and nail polish (NP) conductive ink have emerged as a promising alternative [16–18]. In this composition, NP stands out as an accessible, widely available binder that provides excellent film-forming ability, fast drying, and strong adhesion, enabling the production of homogeneous and reproducible conductive inks through an uncomplicated fabrication process [19–21]. Interestingly, lab-made SPE devices offer numerous advantages, including ease of fabrication, cost-effectiveness, and disposability, making them suitable for large-scale analysis and in-situ applications requiring low sample volumes [22].

The modification of these surface electrodes with nanomaterials, such as metallic and carbon-based nanostructures, is an effective approach to enhance analytical performance in terms of sensitivity and selectivity, which is valuable for the determination of emerging contaminants in complex matrices, such as environmental waters [23–26]. These materials exhibit electroanalytical applicability due to their ability to enhance conductivity, increase surface area, and improve the electrochemical sensing of pollutants [27–29].

Nickel-based nanomaterials, such as metallic nickel nanoparticles (NiNPs), nickel hydroxides (NiOOH), and nickel oxides (NiO), have garnered significant attention owing to their remarkable electrocatalytic properties [30]. These features are primarily associated with their ability to facilitate charge-transfer processes, their high density of acidic and basic active sites, and their highly reactive surface area. Consequently, such materials are well-suited for use as catalysts in a variety of chemical transformations [31] and for the development of high-performance electrochemical sensors [32].

In the literature, innovative buckyball-like nanoarchitectures composed of carbon nanotubes decorated with fullerene-type nanoparticles derived from carbon quantum dots (Cdots) doped with metallic Ni, NiO, and NiOOH have been reported [30]. Additionally, dos Santos et al. [33] proposed the surface modification of a GCE via drop-casting of a hybrid material composed of Cdots/Ni nanoarchitectures for the direct detection of ketoconazole in various matrices. Cdots are a class of carbon-based nanoparticles with sizes below 10 nm and nearly spherical morphology [34]. Structurally, they typically exhibit amorphous to nanocrystalline cores, in which carbon–carbon bonds may present different hybridization states depending on the synthetic route employed. This structural versatility promotes the formation of a variety of surface functional groups, including carboxyl, hydroxyl, and heteroatom-containing moieties, which facilitate the anchoring and stabilization of metallic nanoarchitectures [34, 35]. In this regard, Souza et al. [36] reported the use of Cdots as stabilizing platforms for the fabrication of copper nanoarchitectures aimed at electrochemical sensing applications.

In this context, the present study reports the development of an affordable electrochemical sensor fabricated in-house using flexible acetate sheets as the substrate and a conductive ink based on Gr and NP incorporating nickel nanoparticles, denoted as SPCE-Gr/NiNPs, for the determination of the MFA in environmental samples, such as tap and river water. Although the combination of electrodes printed with metallic nanoparticles has already been explored, the proposed approach integrates the modifying agent (NiNPs) directly into the conductive ink during its preparation, ensuring improved homogeneity and scalability. Unlike conventional strategies based on drop-casting for surface modification, which often result in non-uniform films due to particle aggregation and the “coffee-ring” effect (i.e., the accumulation of suspended particles at the droplet edges during solvent evaporation), compromising reproducibility and hindering large-scale fabrication [37, 38], our approach favors the production of consistent, disposable, and low-cost SPE devices. Notably, the proposed fabrication strategy combines minimal sample consumption (50 µL), suitable analytical performance, and applicability to environmental water samples, supporting its use as a practical alternative for decentralized electrochemical monitoring of MFA.

## Experimental

### Reagents and apparatus

All reagents used in this study were of analytical grade. MFA (≥ 98% wt.) was sourced from Sigma-Aldrich (Germany) and ethanol (99.5% wt.) from Vetec (Brazil). Monobasic (98.0% wt.) and dibasic (99.0% wt.) sodium phosphates, hydrochloric acid (37.0% wt.), and sodium hydroxide (99.0% wt.) from Sigma-Aldrich (Germany) were used for the preparation of buffer solutions. Potassium hexacyanoferrate (III) (98.0% wt.) and potassium chloride (99.5% wt.), sourced from Sigma-Aldrich (Germany), were used to prepare the redox couple solution. Ultrapure water with a resistivity of 18.2 MΩ·cm was obtained using a Merck Milli-Q Reference system. The SPCE-Gr was fabricated using Gr (particle diameter < 20 μm, 99.0% wt.) from Sigma-Aldrich (Germany) and NP (obtained from a local drugstore). The synthesis of NiNPs was carried out using the following reagents: perfluoro-1-butane-sulfonyl fluoride (PFAS) from Sigma-Aldrich, potassium hydroxide (85.0% wt.) from Synth, n-propanol (99.5% wt.) from Neon, and nickel acetate (98.0% wt.) from Synth.

Electroanalytical experiments were performed using a potentiostat/galvanostat model PGSTAT 204 (Metrohm, Switzerland) controlled using the software NOVA version 2.1. A JEOL 7800 F prime electron microscope operating at 30 kV was used to acquire scanning electron microscopy (SEM) micrographs. The samples were dropped onto a Cu/Zn grid. High-resolution transmission electron microscopy (HR-TEM) micrographs were obtained using a JEOL JEM-2100. The samples were prepared for CF300-Cu-50 Carbon Film, 300 Mesh. Raman spectra were obtained on a silicon wafer using a 532 nm laser (1.8 mW) equipped in a WITec alpha 300 R confocal Raman microscope. X-ray diffraction (XRD) has been acquired by a Rigaku diffractometer to investigate the structural characteristics of the NiNPs nanostructures using a monochromatic Cu-Kα source (λ = 0.154 nm) at scanning angle (2θ) in the range of 5° to 90°. In addition, spectrophotometric measurements were performed using an Evolution 300 spectrophotometer (Thermo Scientific, England), operating in scanning mode within the 235–400 nm wavelength range, using a quartz cuvette with a 1.0 cm optical path length. Absorbance was monitored at 235 nm. These experiments were conducted to provide complementary validation of the electrochemical results.

### SPCE-Gr/NiNPs preparation

The NiNPs were obtained by directly mixing 8 mL of F, S-Cdots, at a concentration of 8 mg L^− 1^, with 50 mL alcoholic solution of nickel acetate, after heating at 50 °C for 4 h, as previously reported in another study [30]. The mixture was stirred for 48 h until the green color of nickel acetate turned yellowish, indicating the formation of NiNP**s** functionalized with F, S-Cdots (hereafter referred to as NiNPs). In this procedure, two NiNPs species, NiNPs metallic and Ni(OH)_2_, were obtained as reported by our research group in another study [33]. Conductive control ink was prepared using a mass ratio (1:1) between Gr and NP, according to Silva et al. [16]. The NP plays a crucial role in the formulation of the conductive ink, acting as a binder that enables the effective dispersion and integration of conductive particles, promotes strong adhesion of the ink to the substrate, and provides mechanical stability to the device, as reported in previous work [39]. The conductive control ink (Gr–NP), used to fabricate the unmodified electrode (SPCE-Gr), was prepared by mixing 500 mg Gr with 500 mg NP. On the other hand, the modified ink (Gr/NiNPs) was obtained through an additional step involving the incorporation of 500 µL of NiNPs into the Gr-NP-based formulation ink. For both inks, 1000 µL of propanone was used as the dispersion solvent to ensure proper homogenization.

**Scheme 1:**
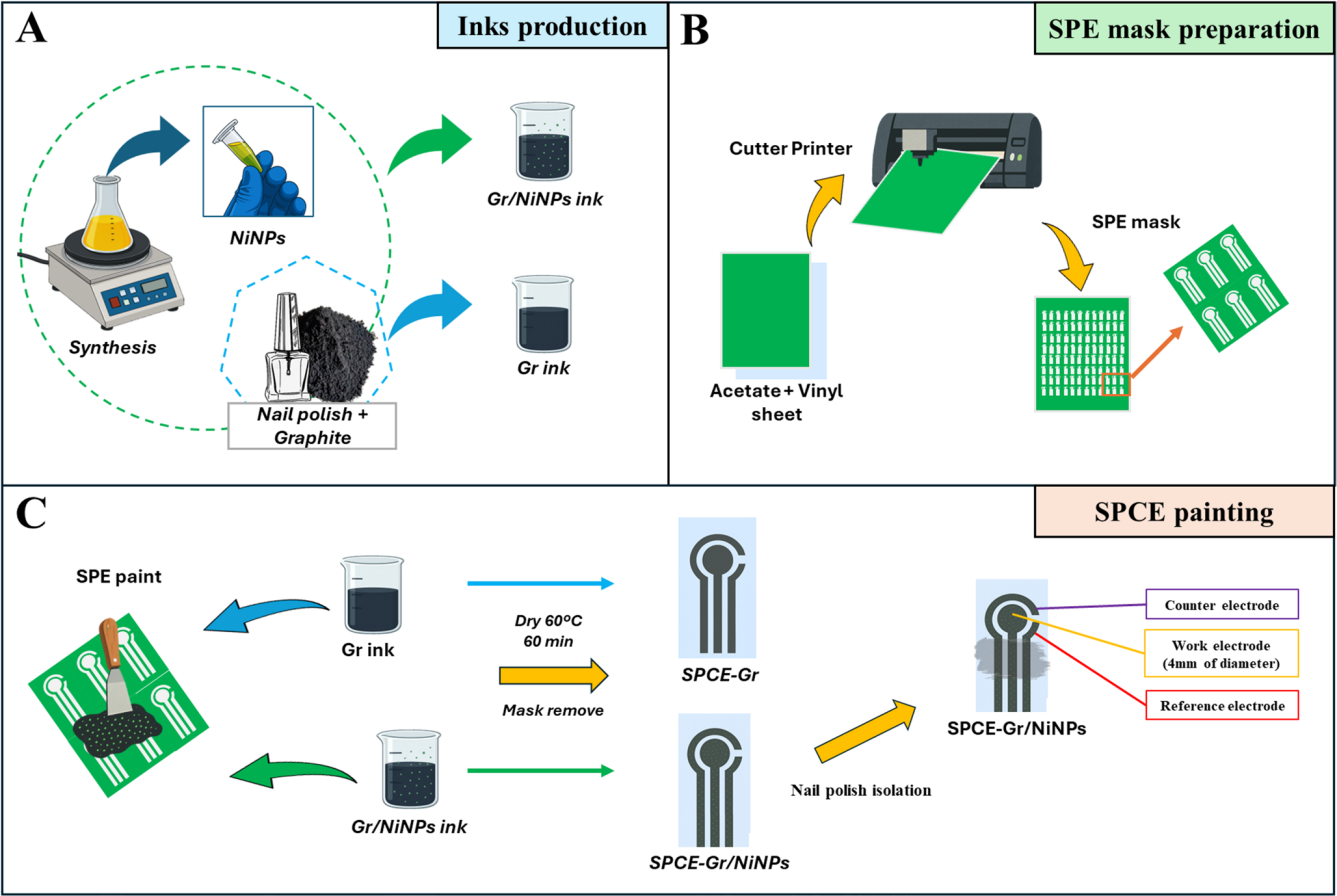
(**A**) Schematic representation of the production process of the conductive inks based on Gr/NP and Gr/NiNPs; (**B**) SPE mask preparation using acetate substrates, vinyl adhesive, and a cutter printer; (**C**) SPCE paint and design of the electrochemical sensors (SPCE-Gr and SPCE-GR/NiNPs)

The SPCEs were produced using a stencil-based fabrication procedure. Initially, a vinyl adhesive film was laminated onto an A4 transparency acetate sheet and patterned with a cutting plotter to define the electrode layout. After removing the patterned vinyl sections to form the stencil, carbon-based ink (either Gr or Gr/NiNPs) was deposited onto the exposed areas. The printed substrate was then dried in an oven at 40 °C for 40 min to promote solvent evaporation and proper formation of the conductive film. Once the curing step was completed, the remaining vinyl stencil was peeled off, and the individual electrodes, SPCE-Gr (unmodified) and SPCE-Gr/NiNPs (modified), were carefully separated. Finally, a thin layer of NP was applied over a defined region of the conductive tracks responsible for electrical contact with the potentiostat, to delimit the active areas of the electrodes. In both devices, the reference electrodes are composed of their respective inks. The possible influence of NiNPs on the potential of the reference electrode was carefully evaluated. However, the voltammetric results showed no significant shift in the reference potential, indicating negligible interference. Scheme 1 illustrates a pictorial representation of the SPCE-Gr and SPCE-Gr/NiNPs fabrication processes.

### Preparation of the real samples

The applicability of the SPCE-Gr/NiNPs sensor for real environmental samples analysis was evaluated by determining MFA in tap and river water using the standard addition method. Tap water samples were initially analyzed without the addition of MFA (sample A), and no detectable signal corresponding to the analyte was observed, indicating the absence of contamination. Subsequently, known amounts of MFA standard solution were spiked into the tap water samples to obtain final concentrations of 1.0 and 5.0 µmol L⁻¹ (samples B and C, respectively). Similarly, river water samples were first analyzed without fortification (sample D), and no detectable MFA signal was observed, suggesting the absence of the analyte in the collected samples. Thereafter, river water samples were fortified with MFA standard solution to final concentrations of 1.0 and 5.0 µmol L⁻¹ (samples E and F, respectively) and subjected to electrochemical analysis. All real samples were prepared in 0.1 mol L⁻¹ PBS, pH 7.0, to ensure appropriate ionic strength and pH conditions.

## Results and discussions

### Material characterization

#### Morphological and structural characterizations

**Fig. 1:**
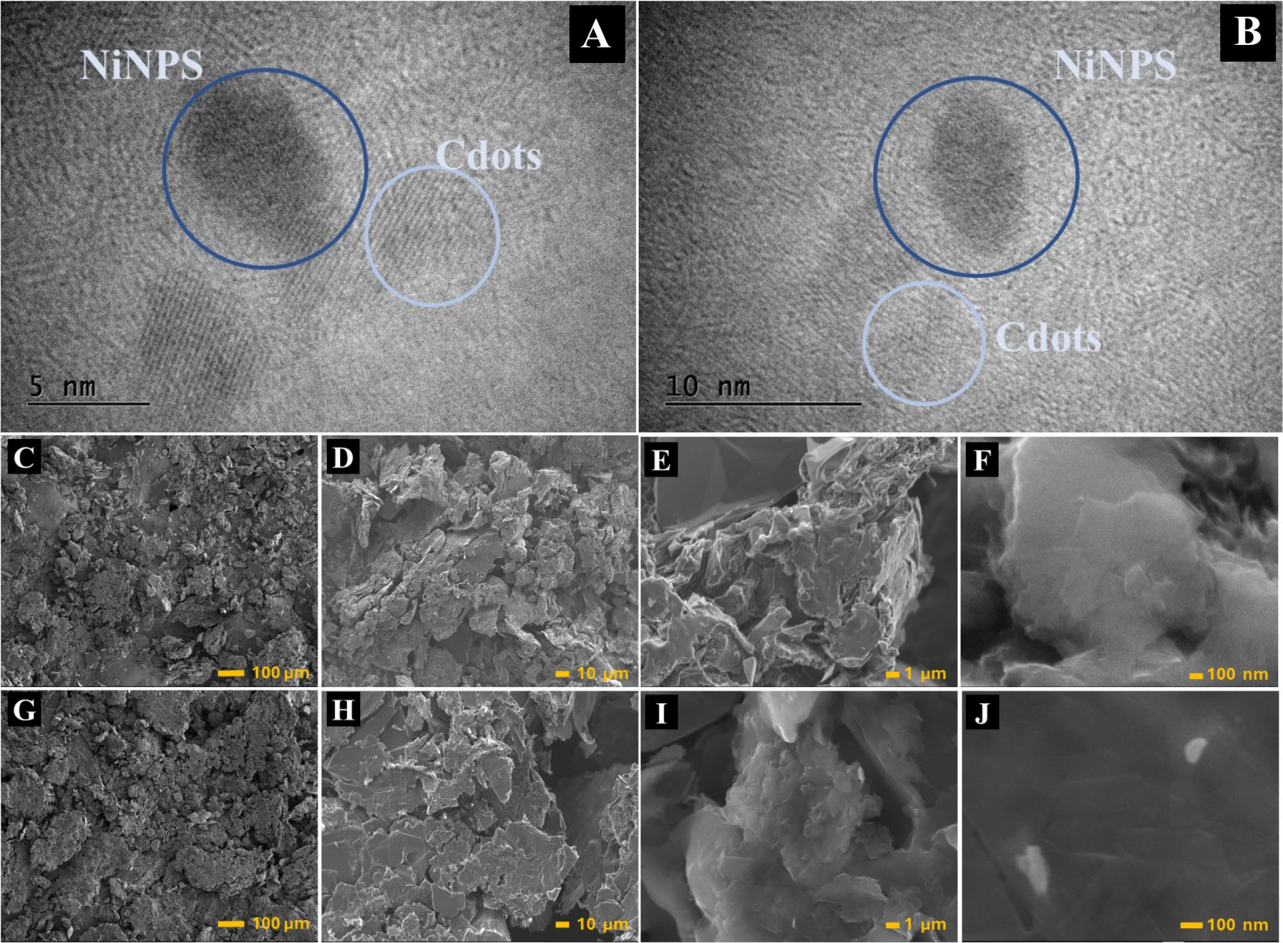
(**A**–**B**) HR-TEM images of the NiNPs. (**C**–**F**) SEM micrographs of SPCE-Gr and (**G**–**J**) SPCE-Gr/NiNPs recorded at different magnifications, ranging from 100 μm to 100 nm

The formation of NiNPs was confirmed by HR-TEM, as shown in Fig. 1A and B. The nanostructures exhibit a quasi-spherical morphology with an average diameter of 5.2 nm, as determined from the particle size distribution (Fig. S1). In addition, an interplanar spacing of 0.217 nm was observed, which corresponds to the (111) lattice planes of nickel nanoparticles [32]. The functionalized F, S-Cdots can also be seen on the side of the NiNPs, with an interplanar spacing of 0.31 nm.

Additionally, SEM images were used to characterize the SPCE devices, composed of Gr/NP, and the Gr/NiNPs, as shown in Fig. 1C-J. Micrographs at different magnifications (Fig. 1C–F) revealed that the conductive ink prepared solely from NP and Gr exhibits a heterogeneous particle size distribution, a rough surface morphology, and low compaction. In contrast, the micrographs corresponding to the Gr/NiNPs-based ink (Fig. 1G–J) showed a more compact and organized structure, with larger and more densely packed domains. This difference is likely associated with a synergistic interaction between graphite and nickel nanoparticles, which enhances material compaction and may improve the electrochemical properties of the electrode by increasing the number of active sites.

The elemental composition and spatial distribution of the materials were investigated by EDS analysis and elemental mapping, as shown in Fig. S2A–D. EDS spectrum of SPCE-Gr (Fig. S2A) exhibits a dominant peak at approximately 0.27 keV, corresponding to carbon (C), confirming that Gr is the main constituent of the material. Additional signals assigned to oxygen (O, ~ 0.52 keV) and sulfur (S, ~ 2.3 keV) are also observed. The presence of oxygen can be attributed to residual oxygen-containing functional groups on the graphite surface or to adsorbed species formed during the fabrication process. The sulfur signal is likely associated with components of the SPCE substrate or residual species from the manufacturing process. The relatively high intensity of the carbon peak compared to the other elements indicates the predominance of the Gr matrix in the unmodified electrode.

After modification with NiNPs, significant changes in the elemental profile are observed. The EDS spectrum of SPCE-Gr/NiNPs (Fig. S2B) shows, in addition to the C, O, and S signals, a distinct peak attributed to Ni, typically detected at ~ 0.85 keV. The appearance of this peak confirms the successful incorporation of NiNPs onto the Gr-modified electrode surface. Although carbon remains the most intense signal, as expected for a Gr-based substrate, the presence of Ni clearly demonstrates the formation of the composite material.

**Fig. 2:**
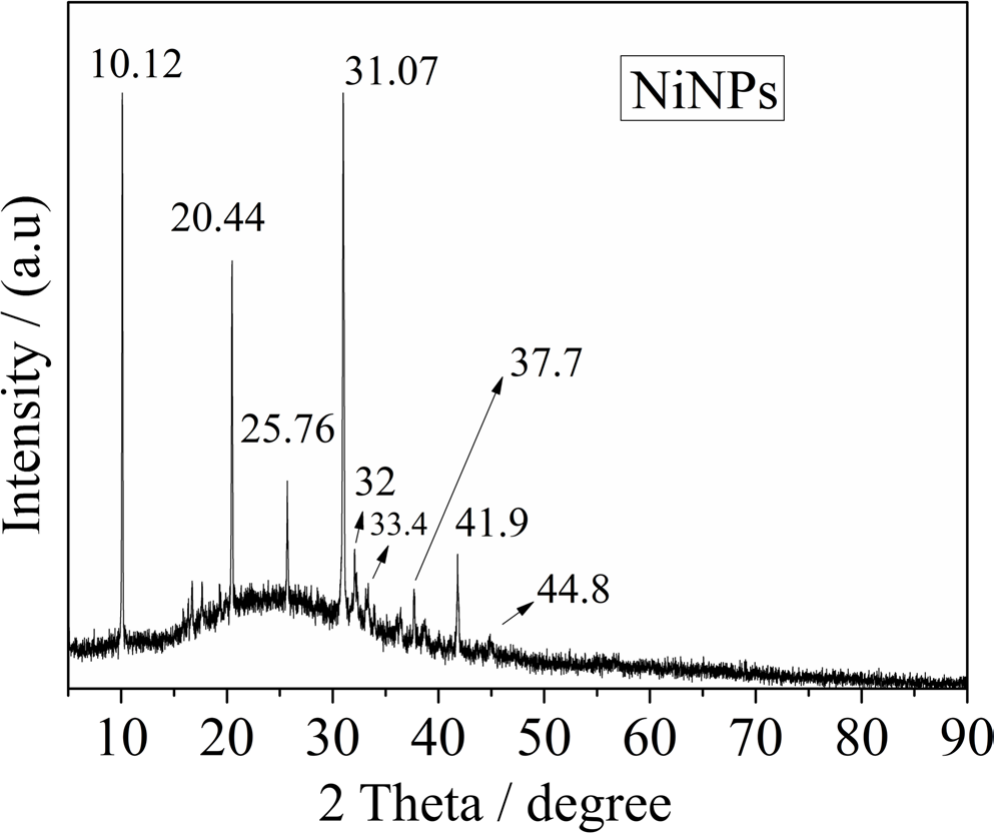
XRD acquired for NiNPs structures

The SEM image and corresponding elemental maps of SPCE-Gr (Fig. S2C) reveal a homogeneous distribution of carbon across the electrode surface, with oxygen uniformly dispersed at lower intensity. No metallic elements are detected, corroborating the EDS spectrum shown in Fig. S2A. In contrast, the SEM image and elemental maps of SPCE-Gr/NiNPs (Fig. S2D) clearly show the presence of Ni distributed over the Gr matrix. The overlap map (C/O/Ni/F/S) indicates that Ni is well dispersed throughout the surface, without evidence of large aggregates at the analyzed scale. This relatively uniform dispersion suggests effective nucleation and anchoring of NiNPs on the Gr layers. It confirms the formation of NiNPs, which could not be clearly identified by SEM due to its inherent resolution limitations, as shown in Fig. 1 (G–J). It is well established that SEM does not have the high resolution to obtain images of nanoparticles due to having a smaller energy source compared to HR-TEM. The enhanced F and S signal in the elemental maps for the SPCE/NiNPs refers to the F, S-Cdots composition. Overall, the EDS spectra and elemental mapping analyses confirm the successful fabrication of the SPCE-Gr/NiNPs composite and demonstrate the effective incorporation and distribution of NiNPs on the Gr-modified electrode surface, supporting its potential application in electrochemical sensing and related fields.

Raman spectroscopy was used to verify the synergistic effect and interaction between Gr and NiNPs, as can be seen in Fig. S3. SPCE-Gr (red line) exhibit the bands around 1356 and 1587 cm^− 1^ correspond, respectively, D band (A1g + E1g + E2g) that refers to disordered graphite structures due to the presence of defects (C–C sp^3^) and G band (A1g + E1g) that refers highly oriented graphite structures (C–C sp^2^) and the bands around 2716 cm^− 1^ refer to the overtone (2D) [40]. The blue line refer interaction between the SPCE-Gr and NiNPs materials mixing, as evidenced by the displacement of the D, G, and overtone bands compared to the ink without NiNPs, in addition to the appearance of bands around 2000, 2437, and 3226 cm^− 1^, referring to the presence of NiNPs structures [41].

Additionally, XRD was performed for the structural characterization of NiNPs (Fig. 2). As observed, the peaks at 2 theta values of approximately 10.12°, 20.44°, and 25.76° are attributed to crystalline carbon dot-related planes and functionalized C-dots structures, likely associated with the presence of abundant surface functional groups [36]. Furthermore, the diffraction peaks located at approximately 31°, 32°, 33.4°, 37.7°, 41.9°, and 44.8° confirm the formation of NiNPs, corresponding to Ni(OH)₂ and NiOx crystalline phases (JCPDS 71-1179, JCPDS 74-2075, and JCPDS 78–0429) [42, 43].

#### Electrochemical measurements

**Fig. 3:**
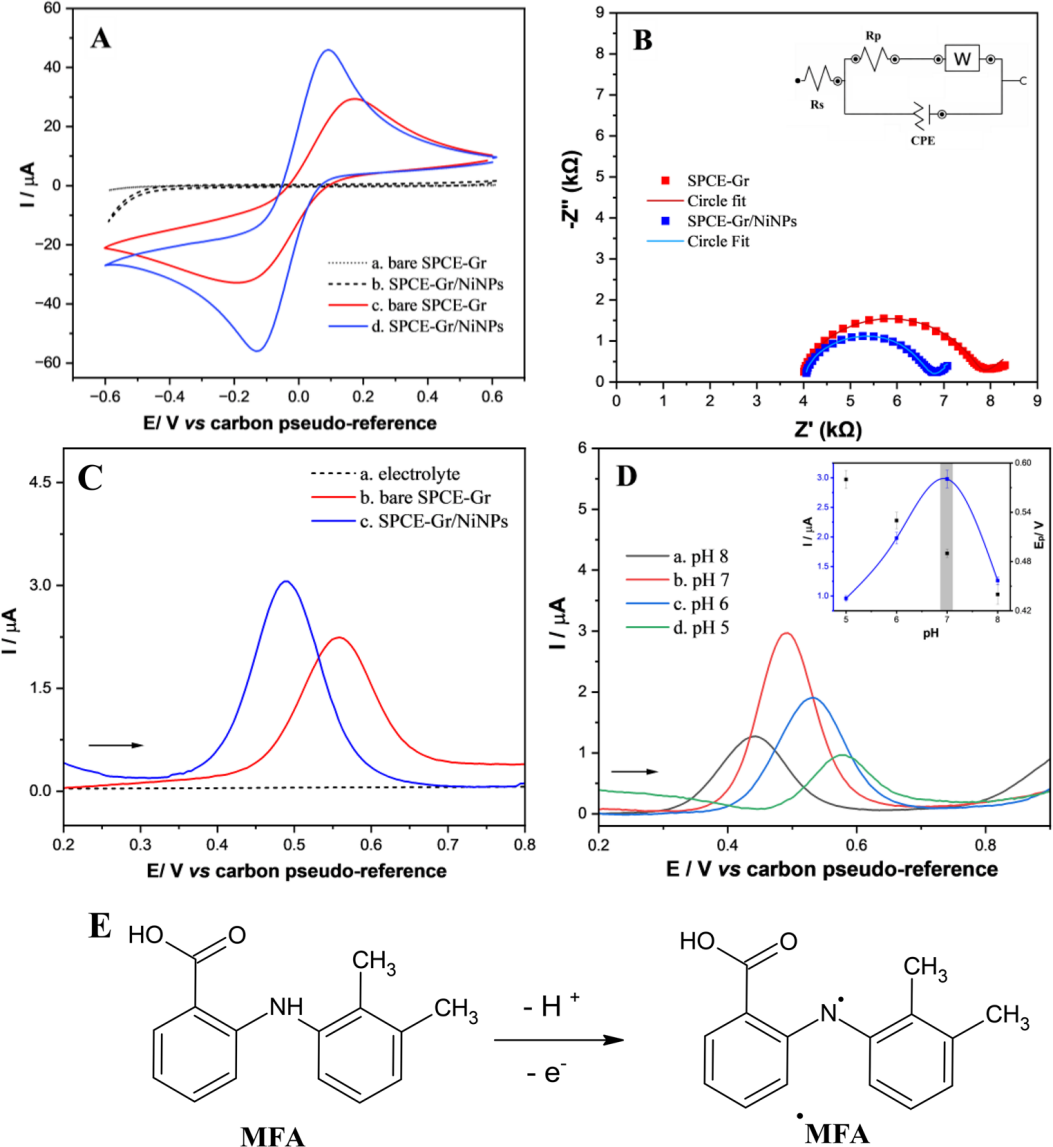
(**A**) CV responses recorded at SPCE-Gr and SPCE-Gr/NiNPs in the absence and presence of [Fe (CN)₆]³⁻ 5.0 mmol L⁻¹, in 0.1 mol L⁻¹ KCl. CV conditions: scan rate of 50 mV s⁻¹, step potential of 2 mV. (**B**) EIS (Nyquist plots) for the [Fe (CN)₆] ³⁻ redox system at SPCE-Gr (red line) and SPCE-Gr/NiNPs (blue line). EIS conditions: frequency range from 100 kHz to 0.1 Hz, amplitude of 10 mV at OCP of 1 mV. (**C**) DPV responses acquired for 10.0 µmol L⁻¹ MFA in 0.1 mol L⁻¹ PBS (pH 7) using both proposed sensors. Initial DPV conditions: step potential of 5 mV, modulation amplitude of 25 mV, modulation time of 50 ms, and scan rate at 10 mV s^− 1^. (**D**) Effect of pH in the response of 10.0 µmol L^− 1^ MFA using PBS 0.1 mol L^− 1^ with pH values between 5 and 8 upon SPCE-Gr/NiNPs. Inset: correlation between I/µA (blue line) and E_p_/V (black line) vs. pH. (**E**) Preliminary electrooxidation mechanism route of MFA

Figure 3A shows the cyclic voltammetry (CV) data recorded in the absence of redox species (dotted lines, blank solution 0.1 mol L⁻¹ KCl) and in the presence of 5.0 mmol L⁻¹ [Fe(CN)₆]³⁻ in 0.1 mol L⁻¹ KCl (solid lines). Figure 3B presents the electrochemical impedance spectroscopy (EIS) results obtained in 5.0 mmol L⁻¹ [Fe(CN)₆]³⁻ containing 0.1 mol L⁻¹ KCl, recorded over a frequency range from 100 kHz to 0.1 Hz, with a perturbation amplitude of 10 mV at the open circuit potential (OCP).

The voltammograms recorded in the supporting electrolyte (0.1 mol L⁻¹ KCl (dotted lines)) exhibit near-zero current throughout the investigated potential window, indicating the absence of significant faradaic processes. The observed response is predominantly capacitive, arising from the charging and discharging of the electrical double layer at the electrode/electrolyte interface. The low current density confirms the electrochemical stability of the electrode material within the selected potential range and the absence of electroactive impurities.

Upon addition of 5.0 mmol L⁻¹ [Fe(CN)₆]³⁻ (solid lines), well-defined anodic and cathodic peaks appear, characteristic of the reversible or quasi-reversible redox process of the [Fe(CN)₆]³⁻/[Fe(CN)₆]⁴⁻ couple. The significant increase in peak currents compared to the electrolyte solution confirms the electrochemical activity of the electrode surface. The peak-to-peak separation (ΔE_p_) and the anodic-to-cathodic peak current ratio provide insight into the electron transfer kinetics. Lower ΔE_p_ values and higher peak currents indicate faster heterogeneous electron transfer and reduced interfacial resistance.

A comparison between the electrodes reveals that the SPCE-Gr/NiNPs exhibit enhanced electrochemical performance relative to the unmodified SPCE-Gr. The modified electrode displays higher peak currents and improved voltammetric profile, suggesting an increased electroactive surface area and improved interfacial conductivity. This behavior can be attributed to the synergistic effect between Gr and NiNPs, which enhances electron transfer and promotes more efficient electrochemical communication between the electrode surface and the redox probe. Moreover, the larger integrated charge under the redox peaks further supports the presence of a higher density of electroactive sites.

To support this, the electrochemically active surface areas (ECSA) of the SPCE-Gr and SPCE-Gr/NiNPS were estimated according to the electrode’s capacitance (C_edl_), obtained using CV at a scan rate of 10 to 150 mV s^− 1^ and 3.0 mol L^− 1^ KCl, in non-faradaic regions (-0.2 to 0.4 V). These data were presented in Fig. S4A. Linear correlations were established between the v/V s^− 1^ and the ΔJ/µA cm^− 2^, as shown in Figure S4B. The C_edl/exp_ were calculated in values of 9.95 ± 0.40 and 17.40 ± 0.80 µF cm^–2^ for SPCE-Gr and SPCE-Gr/NiNPs, respectively. Considering that ECSA is directly proportional to the double-layer capacitance, it can be inferred that the modified electrode exhibits a significantly higher electroactive area. In numerical terms, the capacitance ratio (17.40 / 9.95) indicates that the modified electrode presents an ECSA approximately 1.75 times greater than that of the unmodified electrode, which can be attributed to the increased availability of active sites and enhanced surface roughness provided by the modification. These considerations are consistent with a previous study [44].

EIS measurements were carried out to quantitatively evaluate the charge transfer resistance (R_ct_) associated with the redox process. The Nyquist plots (Fig. 3B) display a semicircular region at high frequencies followed by a linear segment at lower frequencies, characteristic of a diffusion-controlled process. The diameter of the semicircle corresponds to the R_ct_. The SPCE-Gr electrode exhibited an R_ct_ value of 3.8 kΩ, whereas the SPCE-Gr/NiNPs showed a lower R_ct_ of 2.7 kΩ.

The reduced semicircle diameter observed for SPCE-Gr/NiNPs indicates a decrease in charge transfer resistance and, consequently, more favorable electron transfer kinetics at the electrode/electrolyte interface. The lower R_ct_ value is consistent with the enhanced peak currents observed in CV, demonstrating strong agreement between both electrochemical techniques. The improved interfacial properties of the SPCE-Gr/NiNPs electrode can be attributed to increased electrical conductivity, larger effective surface area, and facilitated electron transfer pathways provided by the NiNPs. In addition, the supplementary information provides the raw fitting data obtained from the Randles equivalent circuit for both electrodes (Fig. S5), including a detailed quantitative assignment of all circuit elements, namely the solution resistance (R_s_), R_ct_, constant phase element (CPE), and Warburg element (W). Overall, the combined CV and EIS results demonstrate that the incorporation of NiNPs onto the SPCE-Gr significantly enhances the heterogeneous electron transfer of the [Fe(CN)₆]³⁻/ [Fe(CN)₆]⁴⁻ redox probe, confirming the improved electrochemical performance of the modified electrode.

Figure 3C shows the differential pulse voltammetry (DPV) responses for the MFA electrochemical behavior recorded in 0.1 mol L⁻¹ phosphate buffer solution (PBS) at pH 7.0 at a scan rate of 10 mV s⁻¹. The curves correspond to: (a) SPCE-Gr/NiNPs in the absence of MFA (supporting electrolyte, black dashed line), (b) SPCE-Gr (red line), and (c) SPCE-Gr/NiNPs (blue line), both in the presence of 10.0 µmol L⁻¹ MFA. The blank response obtained with SPCE-Gr/NiNPs (curve a) shows no significant faradaic peak within the investigated potential window, confirming the absence of electroactive interferents and demonstrating a low background current under the selected experimental conditions. After the addition of 10.0 µmol L⁻¹ MFA, a well-defined oxidation peak is observed using both electrodes (curves b and c), corresponding to the electrooxidation of MFA. However, the SPCE-Gr/NiNPs (curve c) exhibits a markedly higher peak current compared to the unmodified SPCE-Gr (curve b), demonstrating the beneficial effect of NiNPs incorporation.

The enhanced current response at SPCE-Gr/NiNPs can be attributed to several factors, such as increased effective electroactive surface area (1.75-fold), improved electrical conductivity due to the Gr matrix, and catalytic activity of NiNPs facilitating electron transfer during MFA oxidation. Additionally, a slight shift in peak potential toward lower values would indicate a reduction in overpotential, reinforcing the catalytic role of NiNPs in promoting MFA oxidation. The improved signal-to-noise ratio obtained with SPCE-Gr/NiNPs highlights its superior analytical performance. Overall, the DPV results demonstrate that the SPCE-Gr/NiNPs provides enhanced performance for MFA detection compared to the bare SPCE-Gr, confirming the effectiveness of the nanostructured modification for electroanalytical applications.

The influence of 0.1 mol L^− 1^ PBS in varied pH-values (5, 6, 7, and 8) on the oxidation process of 10.0 µmol L^− 1^ MFA was studied using the DPV technique in the potential range of 0.2 V to 0.8 V, with the voltammograms presented in Fig. 3D. The linear correlations between E_p_/V and pH (R^2^ > 0.994) presented a slope of 49 mV pH^− 1^ (Fig. 3D-inset). In the correlation between peak current (I_p_) and pH, as shown in the DPV responses, the maximum I_p_ value occurs at pH 7, which was selected for subsequent analysis. Considering the Nernst Eq. (1):

1 $$\:E/V=\:-0.0592\frac{m}{n}pH+\mathrm{b}$$

Therefore, the experimental slope, although lower than the ideal theoretical value, suggests the participation of approximately equal numbers of protons and electrons in the MFA electrooxidation process [45, 46]. Based on this and supported by previous studies on fenamate derivatives [47], we propose a preliminary mechanism route, in which the oxidation involves the loss of one electron and one proton, leading to the formation of a radical species (Fig. 3E).

Additionally, the scan rate influence on the electrochemical oxidation of 10.0 µmol L⁻¹ MFA was investigated by cyclic voltammetry over the range of 5–100 mV s⁻¹ (Fig. S6A). As the scan rate increased, the oxidation peak current increased proportionally, indicating a strong dependence of the electrochemical response on the scan rate. A linear relationship was observed between log (I_p_/µA) and log (v/V s⁻¹) (see Fig. S6B), with a slope of 0.880 and a correlation coefficient higher than 0.993. This behavior suggests that the oxidation of MFA is predominantly adsorption-controlled [48]. Fig. S6C shows the correlation between I_p_/µA and v/V s^− 1^, revealing a confirmation of the adsorption-controlled MFA in SPCE-Gr/NiNPs surface [49]. Using Eq. (2), one electron (*n* = 1) was calculated in the electro-oxidation of MFA, considering the electron transfer coefficient (α) equal to 0.5 applied to irreversible processes [50]. According to the data, the preliminary proposed electro-oxidation process is consistent with the mechanism presented in Fig. 3E.

2 $$\:{\mathrm{E}}_{\mathrm{p}\mathrm{a}}-{\mathrm{E}}_{\mathrm{p}\mathrm{a}/2}=\frac{47.7\:\mathrm{m}\mathrm{V}}{{\upalpha\:}\mathrm{n}}$$

#### DPV optimization parameters

The DPV experimental parameters were systematically optimized using univariate optimization to achieve enhanced sensitivity and well-defined oxidation signals for MFA, as shown in Fig. S7. The step potential, modulation amplitude, and modulation time were evaluated due to their direct influence on peak current intensity. A step potential of 5 mV provided the optimal contribution to current intensity (Fig. S7A). Lower step potentials resulted in reduced signal intensity, whereas higher step potentials led to peak broadening and increased background. Similarly, a modulation amplitude of 40 mV yielded the highest and most reproducible peak current without compromising peak shape, while larger amplitudes caused signal distortion and loss of resolution (Fig. S7B). Regarding modulation time, 50 ms was selected as the optimal value (Fig. S7C), as shorter times were insufficient for effective faradaic response, whereas longer times increased capacitive contributions and signal dispersion. The optimized DPV conditions thus ensured improved analytical performance, enabling reliable and sensitive determination of MFA using the proposed electrode (SPCE-Gr/NiNPs).

#### Analytical measurements of MFA

**Fig. 4:**
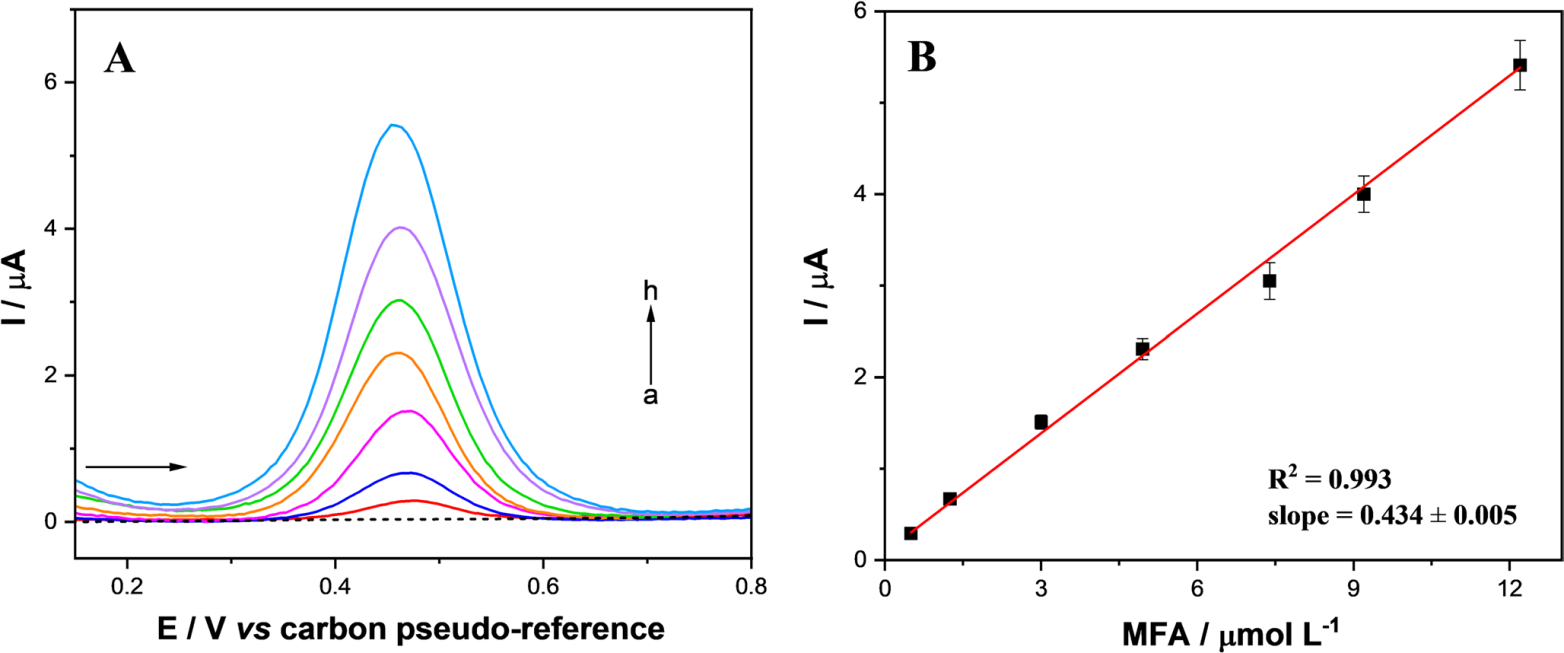
(**A**) DPV responses obtained for MFA at the following concentrations: (a) electrolyte (dashed line); (b) 0.50; (c) 1.25; (d) 3.01; (e) 4.95; (f) 7.39; (g) 9.20; and (h) 12.20 µmol L⁻¹. (**B**) Calibration plot of peak current (Ip, µA) versus MFA concentration (µmol L⁻¹), constructed from the mean values of triplicate measurements (*n* = 3); error bars represent the standard deviation

The electrooxidation of MFA under optimized conditions was performed using different concentration levels by the DPV upon the SPCE-Gr/NiNPs device in the potential range from 0 to 1.0 V (Fig. 3A). All measurements were conducted in triplicate (*n* = 3). Figure 4B presents the linear relationship: I_p_/µA vs. MFA/µmol L^− 1^, expressed by Eq. 3:

3 $$\:{\mathrm{I}}_{\mathrm{p}\mathrm{a}}/\mu\:A=\:0.434\pm\:0.005\:\mathrm{M}\mathrm{F}\mathrm{A}/{{\upmu\:}\mathrm{m}\mathrm{o}\mathrm{l}\:\mathrm{L}}^{-1}+0.087\:\pm\:0.021;\:{\mathrm{R}}^{2}=0.993$$

in the linear range from 0.5 to 12.20 µmol L^− 1^. The relatively narrow linear range can be attributed to the strong adsorptive behavior of the analyte on the electrode surface, which hinders operation at higher concentrations due to the blockage of active sites by oxidation products of MFA. The limit of detection (LOD) was 69.12 nmol L^− 1^, calculated using Eq. (4) and the limit of quantification (LOQ), Eq. (5):

4 $$\:\mathrm{L}\mathrm{O}\mathrm{D}\:=\:3\times\:\frac{{\upsigma\:}}{\mathrm{b}}$$

5 $$\:\mathrm{L}\mathrm{O}\mathrm{Q}\:=\:10\times\:\frac{{\upsigma\:}}{\mathrm{b}}$$

where σ represents the standard deviation of the blank (*n* = 10) and b is the slope of the calibration curve.

Table 1 compares the analytical performance of the proposed SPCE-Gr/NiNPs sensor with previously reported electrochemical platforms for MFA determination. A critical analysis of the literature shows that this determination has predominantly been explored using conventional electrode substrates, particularly GCE and CPE, generally modified with nanostructured or metal-based materials to maximize sensitivity. According to the data presented, the sensors described in the literature exhibit lower LOD values than the proposed platform, and some of them also show wider linear ranges, indicating that, from a strictly analytical standpoint, the SPCE-Gr/NiNPs does not stand out as the most sensitive system among those compared. However, these performances are generally associated with more elaborate modification strategies, involving multi-step synthesis routes, metal oxide doping, or complex nanostructured composites. In contrast, the proposed SPCE-Gr/NiNPs is based on a lab-made SPE platform fabricated through a simple and low-cost procedure, using accessible materials and requiring only 50 µL of sample per analysis. In addition, LOD should not be interpreted as the sole criterion for analytical comparison, since its practical relevance also depends on its relationship with the demonstrated working range. In this regard, some literature reports show considerable discrepancies between the LOD value and the lower measurable concentration level of the linear range, which suggests caution in the isolated interpretation of this parameter. For example, the ZnO NPs/CPE platform presents an LOD approximately 40-fold lower than the first point of the linear range. Thus, the main contribution of the present work does not lie in maximizing analytical sensitivity but in providing a more affordable and operationally uncomplicated SPE device for MFA determination, combining cost-effectiveness, disposability, portability, and minimal sample consumption, which reinforces its suitability for environmental electroanalysis.

**Table 1:** Comparison of SPCE-Gr/NiNPs performance with electrochemical sensors for MFA determination presented in the literature

Electrode	Modifier	Technique	Linear range (µM)	LOD (µM)	Ref
GCE^1^	SWCNT^3^	SWV^4^	0.1–35	0.013	[51]
GCE	CNT^5^	DPV	0.05–0.1 and 0.1–0.7	0.063	[52]
CPE^2^	MWCNTs/RuTiO_2_^6^	SWV	0.01–0.9	0.0045	[8]
CPE	ZnO NPs	DPV	0.02–1.0	0.0005	[53]
CPE	Ag-doped TiO_2_	SWV	0.01–3.0	0.002	[54]
CPE	Cu(II) doped zeolite	DPV	0.3 − 100	0.04	[55]
CPE	Fe (III) complex	DPV	0.02–150.0	0.02	[9]
CPE	Co_3_V_2_O_8_	DPV	0.09–900.0	0.0257	[56]
SPCE-Gr	NiNPs	DPV	0.50–12.20	0.069	This work

^1^GCE: glassy carbon electrode; ^2^CPE: carbon paste electrode; ^3^SWCNT: Single walled carbon nanotubes; ^4^SWV: Square wave voltammetry; ^5^CNT: carbon nanotubes; ^6^MWCNT: multiwalled carbon nanotube ruthenium-doped TiO_2_ nanoparticles loaded into multi-walled carbon nanotubes

#### Reproducibility and stability

Figure S8 evaluates the reproducibility and storage stability of the SPCE-Gr/NiNPs sensor toward 9.0 µmol L⁻¹ MFA using DPV measurements. Figure S8A shows three independently prepared SPCE-Gr/NiNPs electrodes tested under identical experimental conditions. The oxidation peak currents are highly reproducible, with a relative standard deviation (RSD) of 3.85%, indicating excellent fabrication reproducibility and surface homogeneity. The low RSD demonstrates that the screen-printing process, combined with NiNPs incorporation, provides consistent electroactive surface characteristics and reliable analytical response.

Figure S8B presents a stability study over 30 days. The DPV responses recorded at different storage times (days 1, 5, 10, 15, 20, and 30) show only a slight decrease in peak current intensity, while maintaining well-defined and symmetric oxidation peaks. The gradual signal attenuation observed after prolonged storage is likely associated with minor surface passivation or partial oxidation of NiNPs; however, the overall electrochemical activity remains largely preserved. Fig. S8C summarizes the current behavior over time. The proposed sensor maintains 100% ± 4%, 104% ± 6%, and 101% ± 5% of its initial response for day 1, 5, and 10, respectively, indicating excellent short-term stability. After 15 and 20 days, the current retention remains above 90%, and even after 30 days, approximately 88% ± 4% of the original signal is preserved. These results confirm good long-term stability, with minimal degradation of the electrocatalytic surface. Overall, the low RSD and high current retention demonstrate that the SPCE-Gr/NiNPs platform provides reliable, reproducible, and stable performance for MFA determination at 9.0 µmol L⁻¹, reinforcing its suitability for practical electroanalytical applications.

#### Interferences analysis

The selectivity of the SPCE-Gr/NiNPs electrode toward MFA electrooxidation was evaluated by DPV in the presence of potentially interfering species, such as ivermectin (IVE), uric acid (UA), and ascorbic acid (AA), flunixin (FNX), vancomycin (VAN), gentamicin (GEN), indomethacin (IND), and piroxicam (PRX), using a 1:10 ratio between MFA and interfering agent as shown in Fig. S9. The oxidation current obtained for MFA in the absence of interferents was normalized to 100%, and the relative current responses were analyzed under identical experimental conditions (Fig. S9B). The results demonstrate that the presence of common electroactive compounds, including organic acids and pharmaceutical molecules (VAN, GEN, IND, and PRX), induces only minimal variations in the MFA oxidation signal (97.0% ± 4.0% to 107.0% ± 3.0%). Among the pharmaceutical molecules evaluated, the IVE showed an increase in the oxidation signal (107.0% ± 3.0%). In these cases, the current responses remain within a narrow range around the reference value, indicating that the electrochemical process of MFA is substantially unaffected by these coexisting species. Such behavior reflects a high degree of selectivity inherent to the NiNPs-based catalytic interface.

Slight current suppression observed in the presence of UA (82.0% ± 7.0%) and AA (88.0% ± 5.0%) is attributed to competitive adsorption at the electrode surface. However, the limited magnitude of these effects suggests that MFA preferentially interacts with the catalytically active NiNPs sites, allowing its oxidation to proceed efficiently even in the presence of structurally and electrochemically similar interferents, despite their higher concentrations relative to MFA. Overall, the interference study confirms that the SPCE-Gr/NiNPs electrode operates via a selective electrocatalytic mechanism, characterized by a strong affinity for MFA and robust tolerance to common interferents. These findings underscore the suitability of the proposed catalytic system for practical electrochemical applications, particularly in complex matrices where selectivity is a critical performance parameter.

#### MFA determination in real samples

**Fig. 5:**
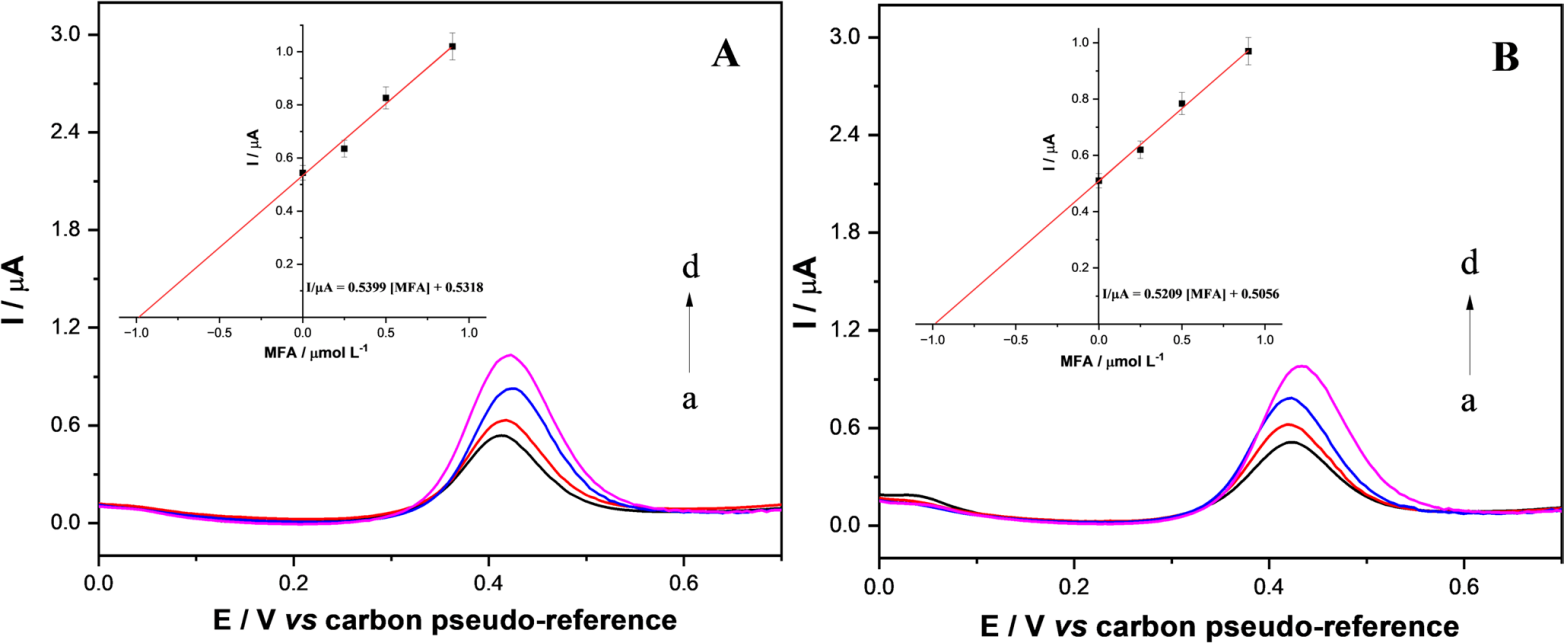
DPV responses recorded for spiked water samples: (**A**) tap water B (black line) at an initial MFA concentration of 1.0 µmol L⁻¹, and (**B**) river water E (black line) at the same concentration level, followed by three successive additions of MFA at 0.25 (red line), 0.50 (blue line), and 1.0 µmol L⁻¹ (magenta line). The corresponding insets show the calibration plots obtained using the standard addition method

The analytical applicability of the proposed SPCE-Gr/NiNPs sensor was further validated through recovery experiments performed in tap and river water samples (see Table 2). All analyses were performed in triplicate. Initially, non-spiked tap water (sample A) and river water (sample D) were analyzed, and no detectable MFA signal was observed, indicating the absence of MFA contamination in these matrices. Thus, recovery studies were carried out by spiking both environmental water samples with known concentrations of MFA (1.0 and 5.0 µmol L⁻¹), a common practice adopted in recent studies for accuracy evaluation [57–60]. Figure 5A and B present the DPV data for the initial analysis of spiked samples (tap water sample B and river water sample E). For tap water samples, the recoveries ranged from 98.0% ± 2.0% to 99.0% ± 3.0%, with experimentally found concentrations (0.98 and 4.95 µmol L⁻¹) in close agreement with the added values. These results indicate the high accuracy of the proposed method and negligible matrix interference in tap water samples. Similarly, river water samples exhibited satisfactory recoveries between 96.0% ± 5.0% and 97.0% ± 3.0% for the tested concentration levels. Despite the greater complexity of river water matrices, the sensor maintained appropriate analytical performance, indicating good selectivity and robustness under real-world sample conditions. Overall, the recovery values (96.0–99.0%) and low relative standard deviations demonstrate that the SPCE/NiNPs platform provides accurate and precise determination of MFA in environmental water samples. Additionally, a statistical comparison using Student’s t-test was carried out between the results obtained by the electrochemical method and those from the reference spectrophotometric method. The corresponding UV–Vis spectra and calibration curve are presented in Fig. S10. The calculated t value (t_calc_ = 1.57) was lower than the critical value (t_crit_ = 3.18), indicating no statistically significant difference between the two methods. These results demonstrate the robustness of the proposed electrochemical approach as an alternative tool for the determination of MFA in environmental contexts.

**Table 2:** Determination of MFA in tap and river water samples using the SPCE-Gr/NiNPs sensor and reference UV-Vis spectrometric method

Samples	Added (µmol L^− 1^)		Found (µmol L^− 1^)	Recovery (%)	Found (µmol L^− 1^)	Recovery (%)
Tap water A	-	No detected		-	No detected	-
Tap water B	1.0	0.98 ± 0.02		98.0 ± 2.0	0.98 ± 0.04	98.0 ± 4.0
Tap water C	5.0	4.95 ± 0.15		99.0 ± 3.0	5.00 ± 0.20	100.0 ± 4.0
River water D	-	No detected		-	No detected	-
River water E	1.0	0.97 ± 0.03		97.0 ± 3.0	0.97 ± 0.050	97.0 ± 5.0
River water F	5.0	4.80 ± 0.25		96.0 ± 5.0	4.90 ± 0.10	99.0 ± 2.0

Student test: t_calc_ = 1.57 < t_crit_ = 3.18 at a 95% confidence level (*p* = 0.215)

## Conclusion

In this work, a novel SPCE-Gr/NiNPs electrochemical sensor was successfully developed for the sensitive determination of mefenamic acid. The incorporation of NiNPs onto the Gr-based SPCE significantly enhanced the electrochemical performance, promoting faster electron transfer kinetics and increasing the effective electroactive surface area. Morphological and structural characterizations demonstrated effective incorporation of the NiNPs into Gr-ink. Electrochemical characterization by CV and EIS confirmed the improved interfacial properties of the modified electrode, evidenced by higher faradaic currents and lower charge transfer resistance compared to the unmodified platform. DPV provided a well-defined and sensitive oxidation response for mefenamic acid, with a linear range from 0.5 to 12.20 µmol L^− 1^ and a limit of detection of 69.12 nmol L^− 1^, demonstrating suitable analytical performance within the studied concentration range. The proposed sensor exhibited good reproducibility (RSD = 3.85%), satisfactory long-term stability (88% signal retention after 30 days), and excellent recovery values (96–99%). In addition, orthogonal validation against a reference spectrophotometric method in tap and river water samples confirmed its accuracy and negligible matrix interference. Overall, the SPCE-Gr/NiNPs platform combines analytical reliability, operational simplicity, low cost, and suitability for on-site analysis. These features highlight its strong potential for practical environmental sensing of MFA.

## Supplementary Information

Below is the link to the electronic supplementary material.

Supplementary Material 1 (DOCX 10.2 MB)

## Data Availability

No datasets were generated or analysed during the current study.
