# Supplementary material for: A novel nail polish/graphite/nickel nanoparticles-based disposable screen-printed sensor for enhanced voltammetric detection of mefenamic acid in environmental water samples
Source: Mikrochim Acta. 2026 Jun 4;193(7):443. doi: 10.1007/s00604-026-08105-4 (PMC13236844; doi:10.1007/s00604-026-08105-4)
Supplement: Supplementary file 1 — Supplementary Material 1 (DOCX 10.2 MB) [file 604_2026_8105_MOESM1_ESM.docx]

**A novel nail polish/graphite/nickel nanoparticles-based disposable screen-printed sensor for enhanced voltametric detection of mefenamic acid in environmental water samples**

Luís Eduardo da Conceição Teixeira^1^, Francisco Walison Lima Silva^1^, Anne Alves Macedo^1^, Octávio P L de Souza^2^, João H A Ferreira^2^, Thiago C Canevari^2^, Ricardo Erthal Santelli^1,3^, Lucas Vinicius de Faria^1^, and Fernando Henrique Cincotto^1,3*^

^1^Departamento de Química Analítica, Instituto de Química, Universidade Federal do Rio de Janeiro, 21941-909, Rio de Janeiro-RJ, Brazil.

^2^Multifunctional nanomaterials hybrid laboratory (LABNAHM), Engineering School, Mackenzie Presbyterian University, 01302-907 São Paulo-SP, Brazil.

^3^ National Institute of Science and Technology of Bioanalytics Lauro Kubota (INCTBio-LK), Campinas-SP, 13083-970, Brazil.

*Corresponding author

E-mail address: fernandocincotto@iq.ufrj.br; fernandocincotto@gmail.com


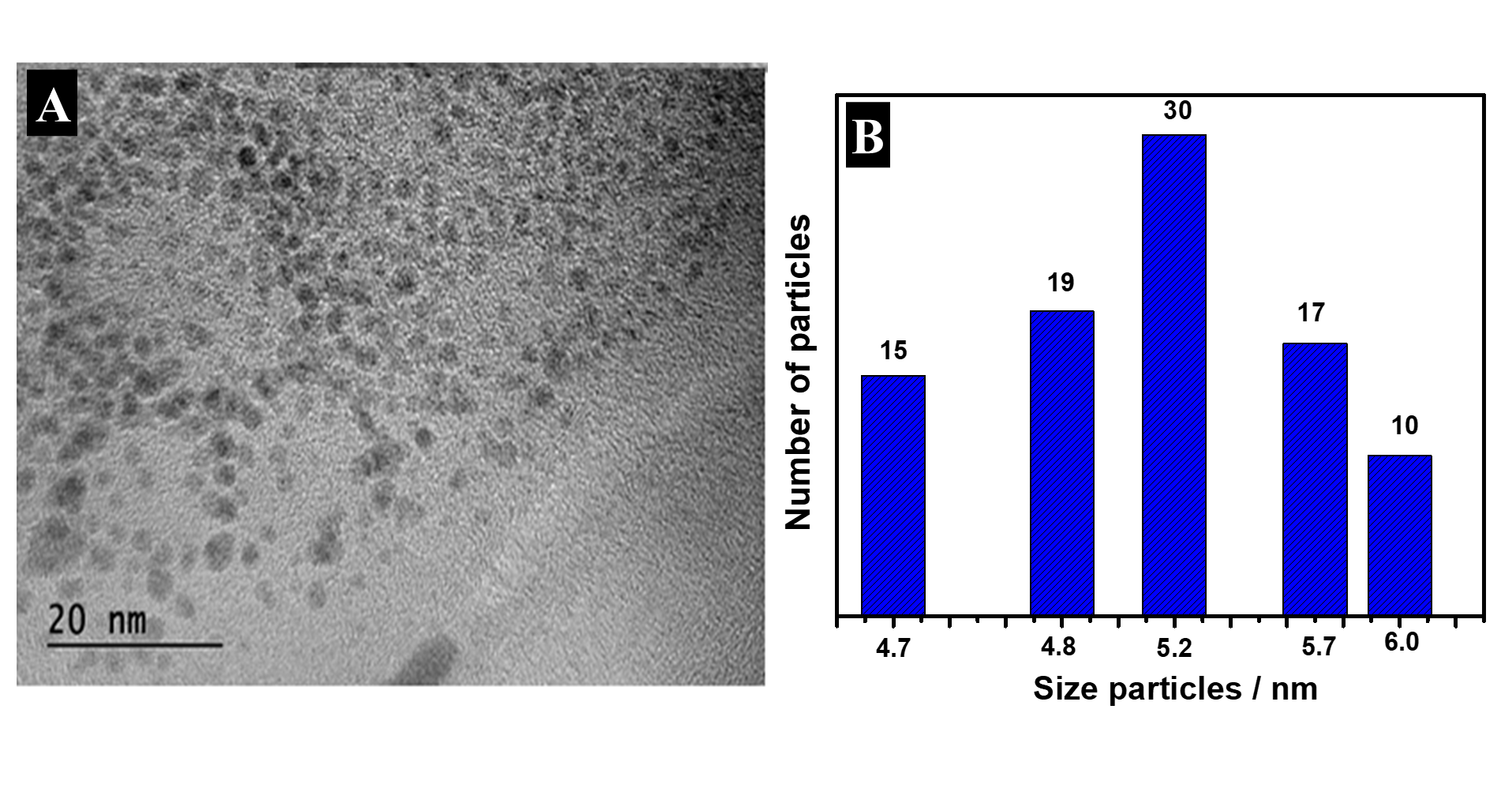


**Figure S1 –** (A) HR-TEM image and (B) corresponding particle size distribution histogram of NiNPs.

**
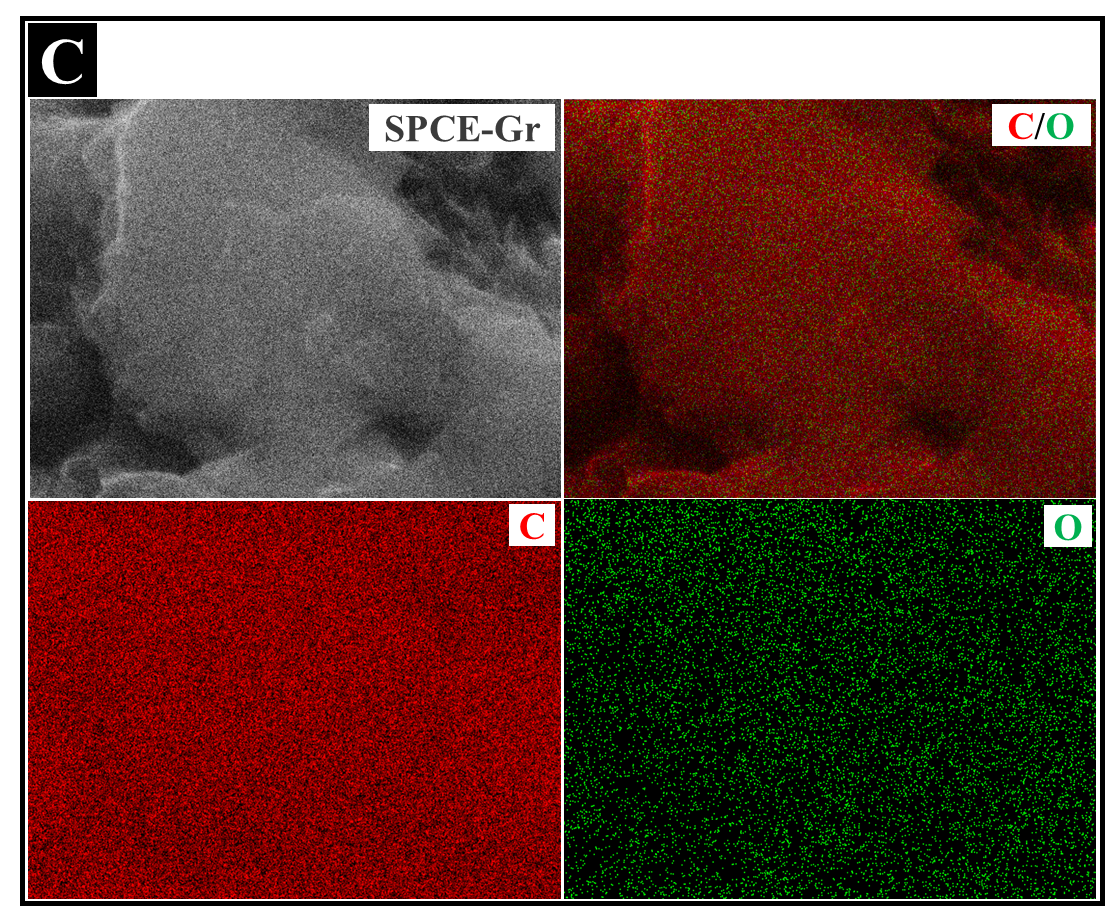

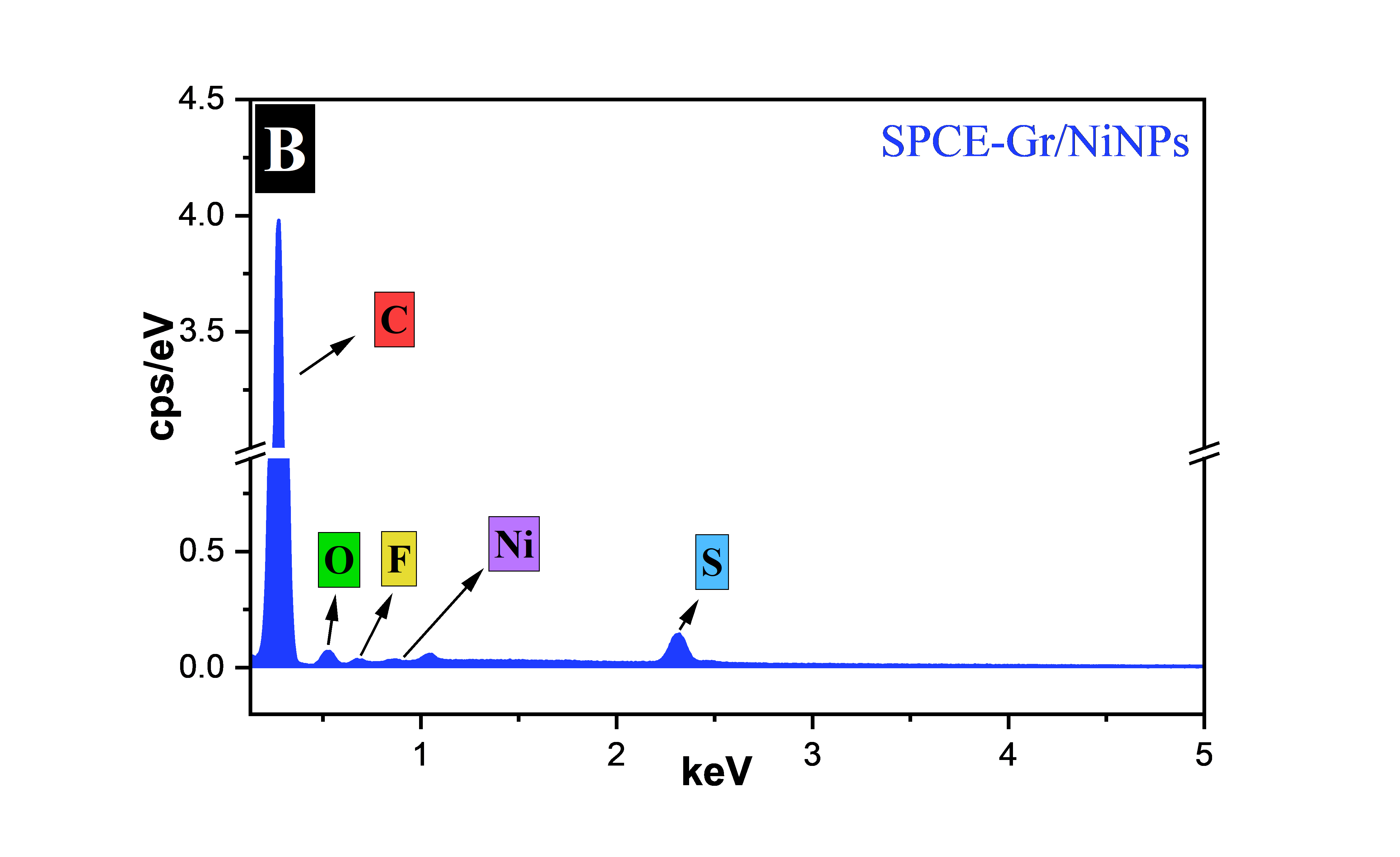

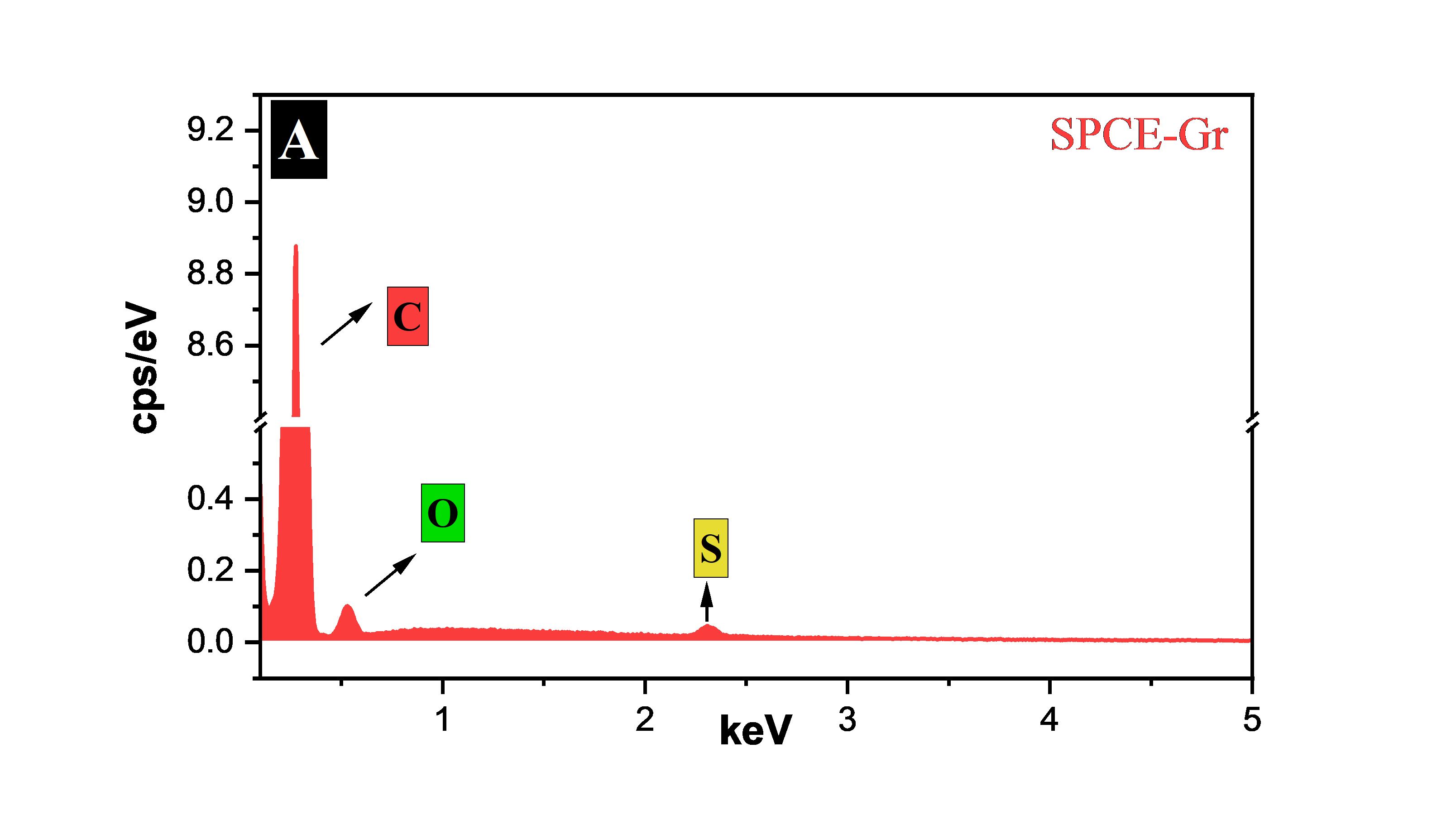

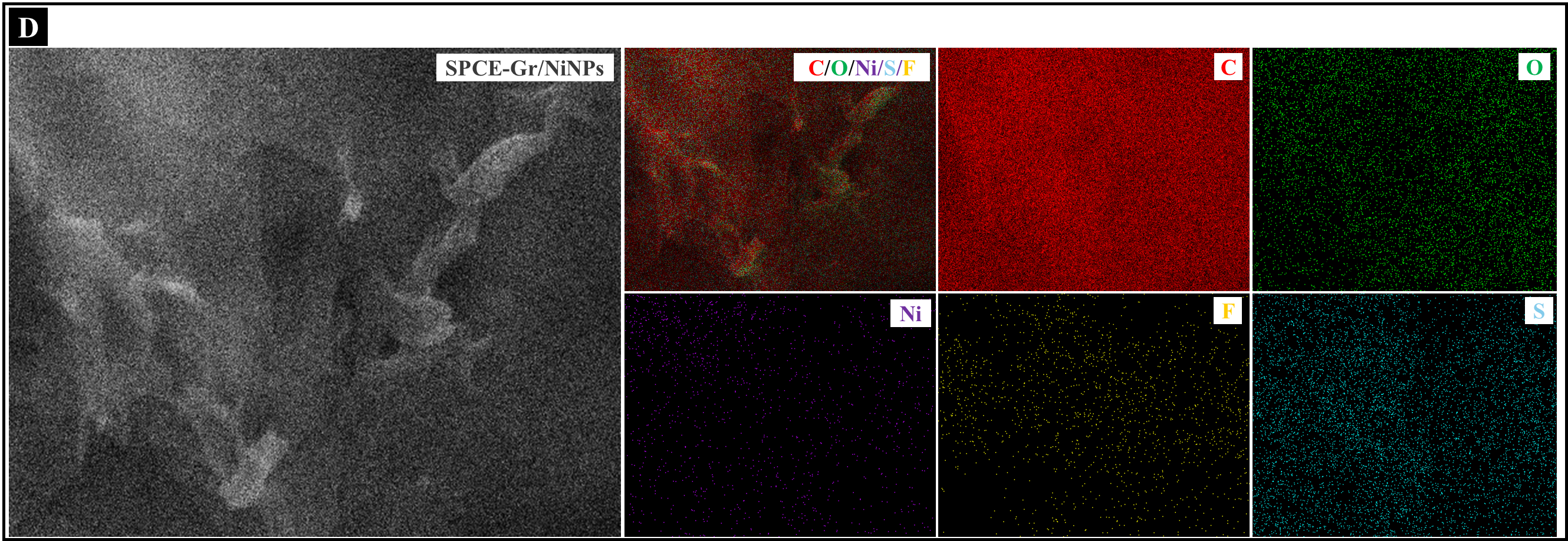
**

**100 nm**

**100 nm**

**Fig. S2 –** EDS analysis and respective EDS mapping analysis for the SPCE-Gr (A and C) and SPCE-Gr/NiNPs (B and D).

**
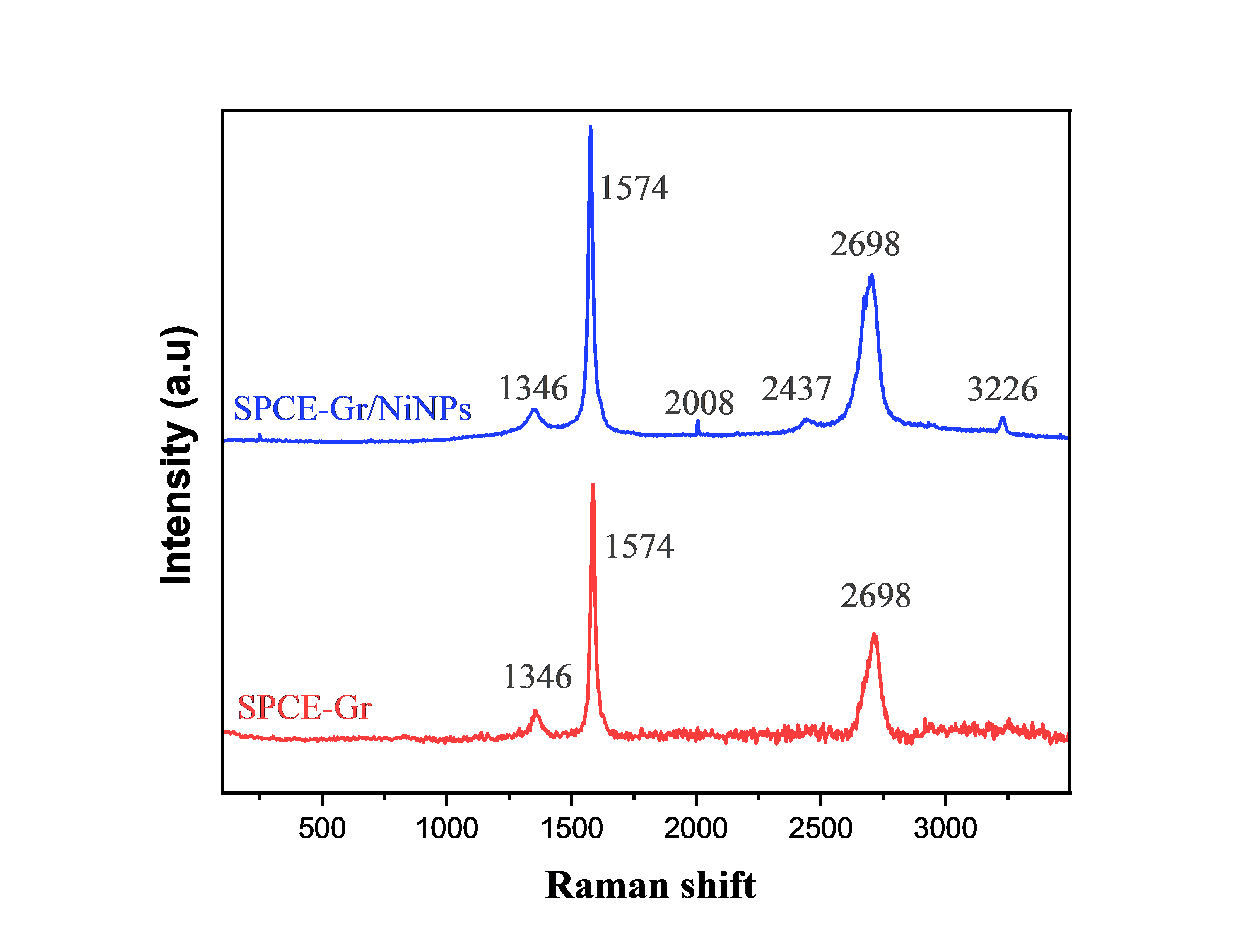
**

**Fig. S3 –** Raman spectra of SPCE-Gr (red line) and SPCE-Gr/NiNPs (blue line).


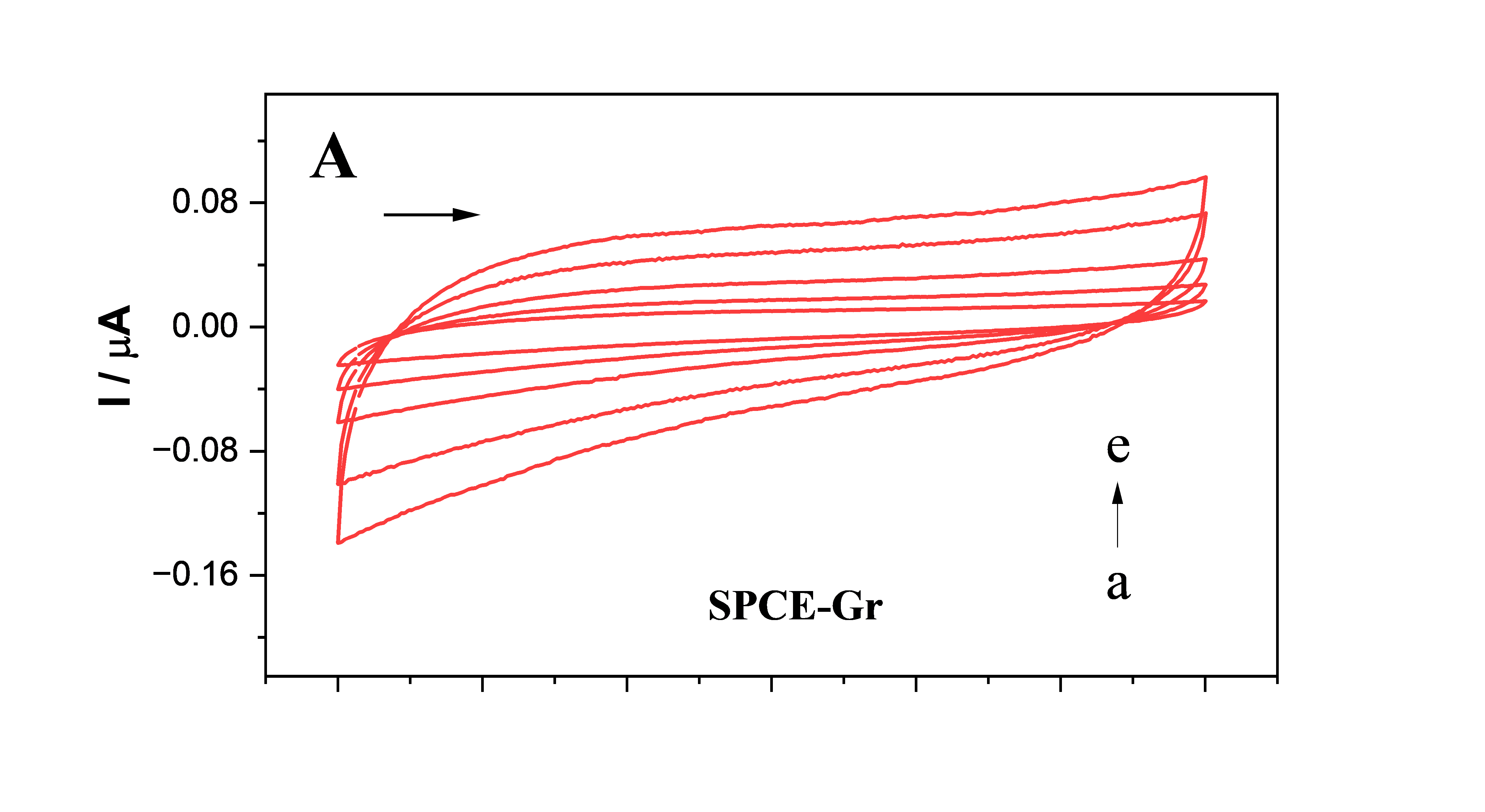

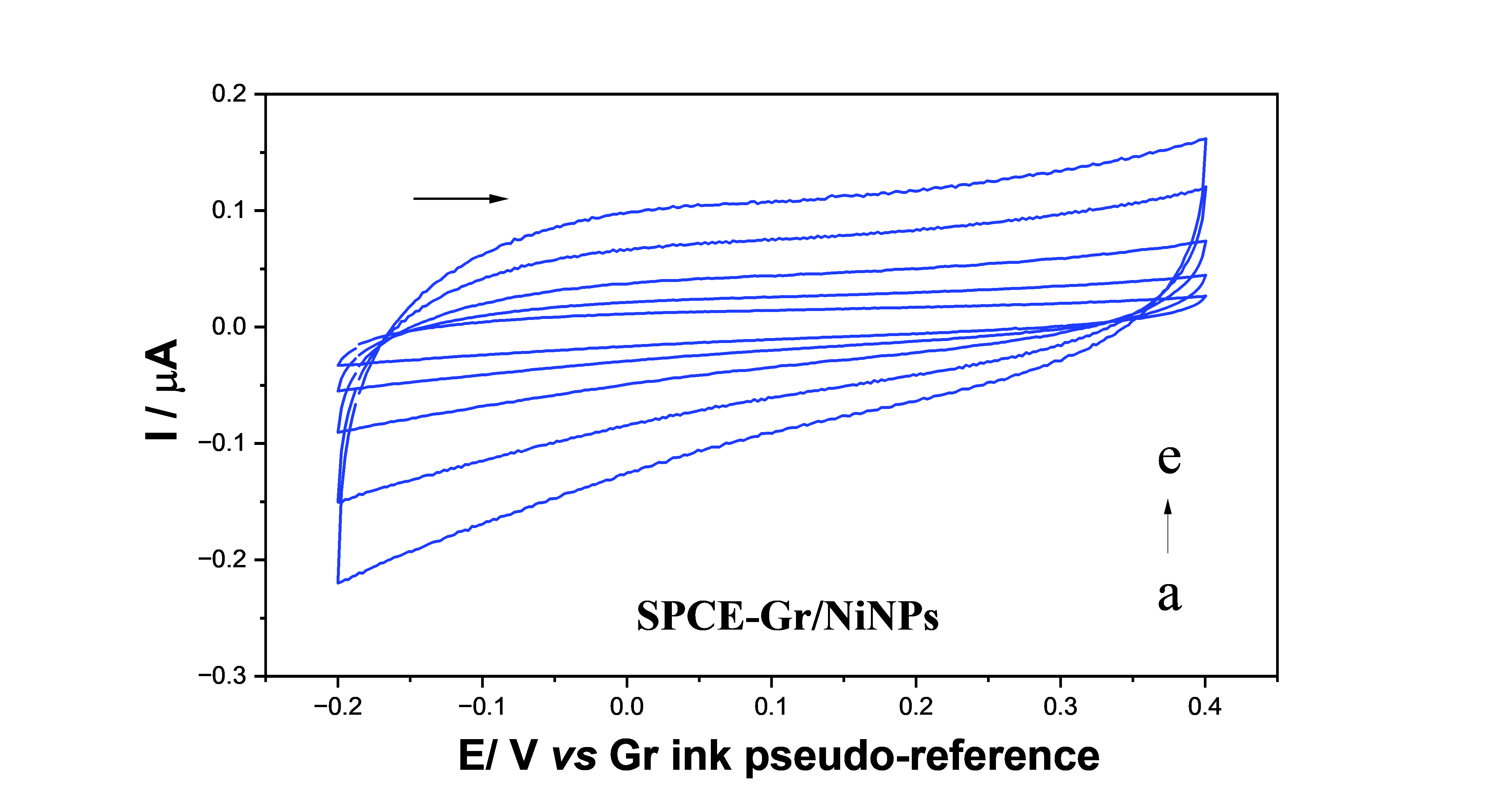

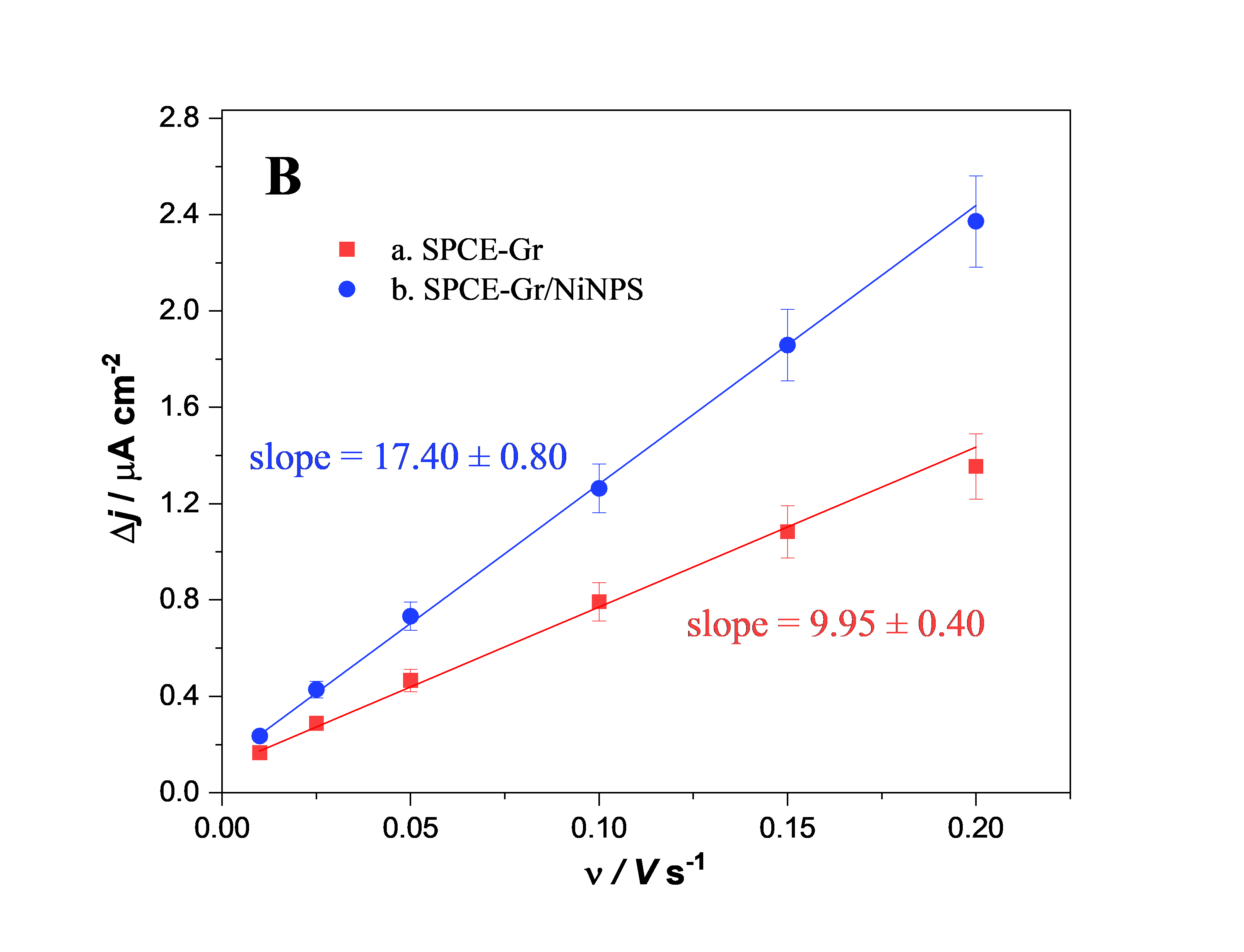


**Figure S4** – (A) CV data obtained in 3.0 mol L^-1^ KCl using the bare SPCE-Gr (black line) and SPCE-Gr/NiNPs (red line); (B) Linear correlation between variation of the current density and scan rates ((a) 10 at (e) 150 mV s^-1^).


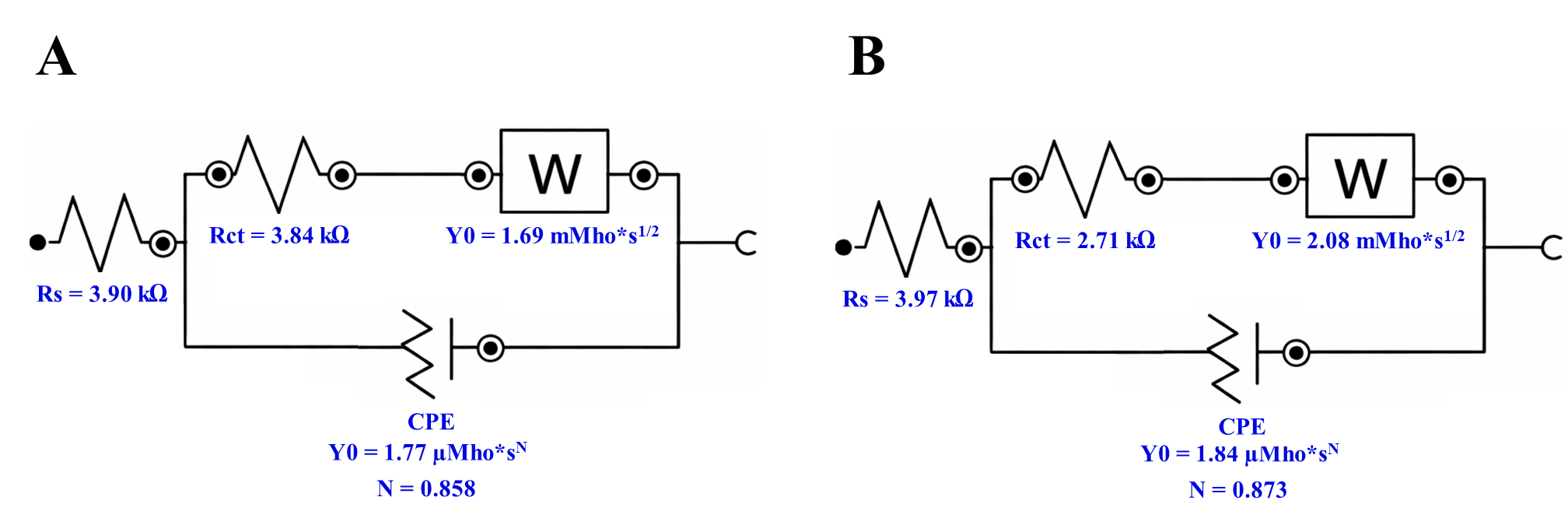


**Fig. S5** – Raw fitting data based on the Randles equivalent circuit for (A) SPCE-Gr and (B) SPCE-Gr/NiNPs, including the quantitative assignment of all circuit elements: solution resistance (R_s_), charge transfer resistance (R_ct_), constant phase element (CPE), and Warburg element (W).


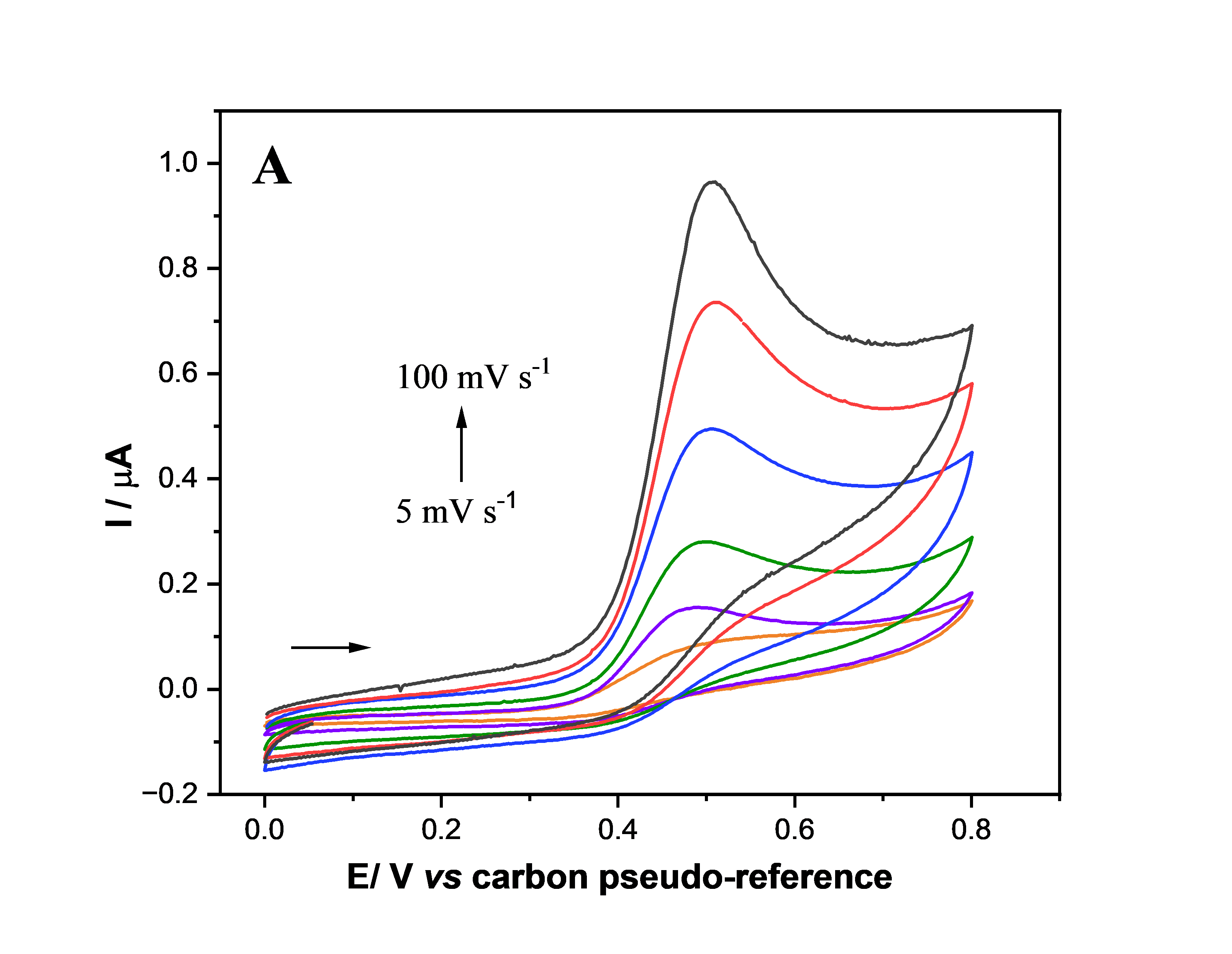

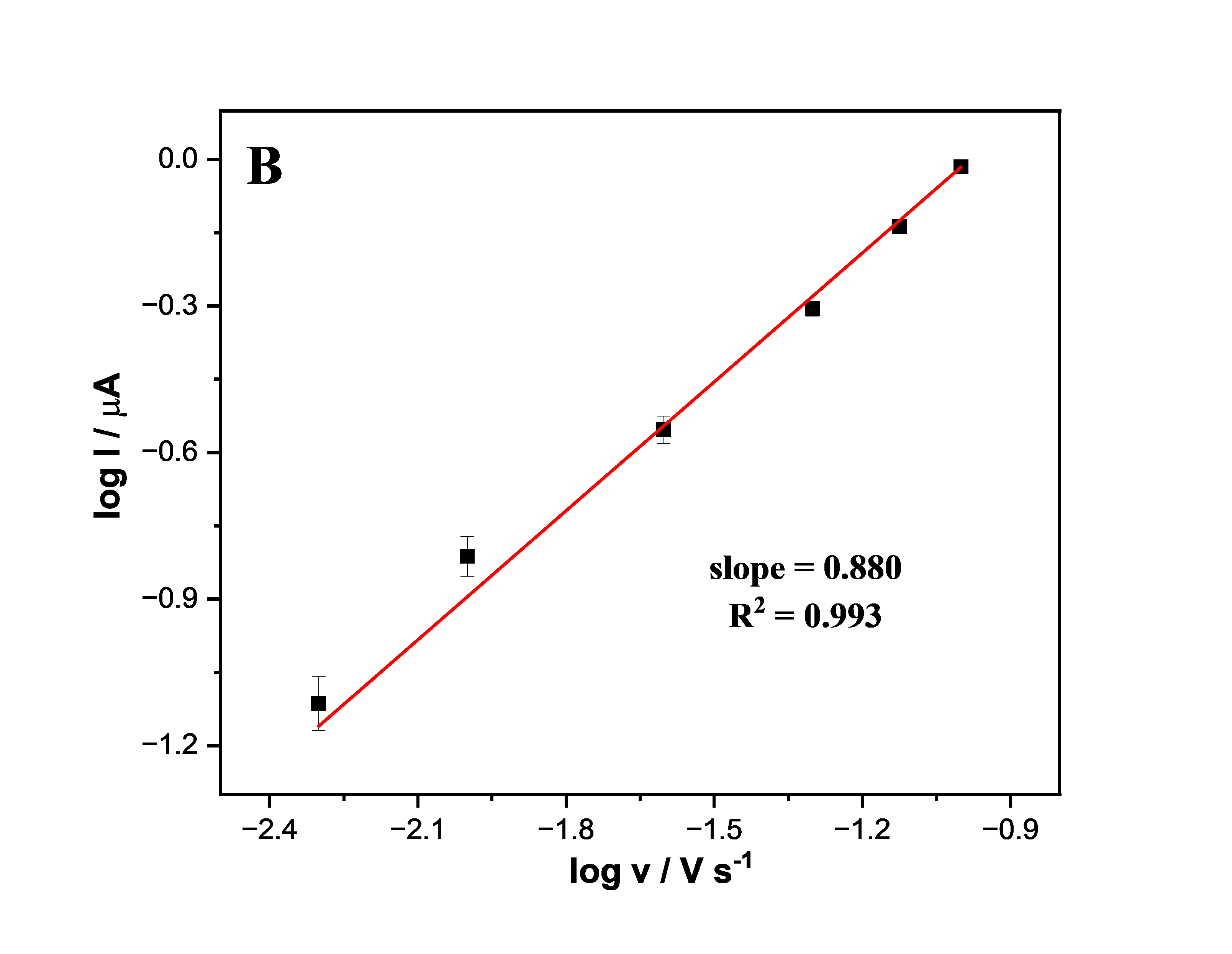

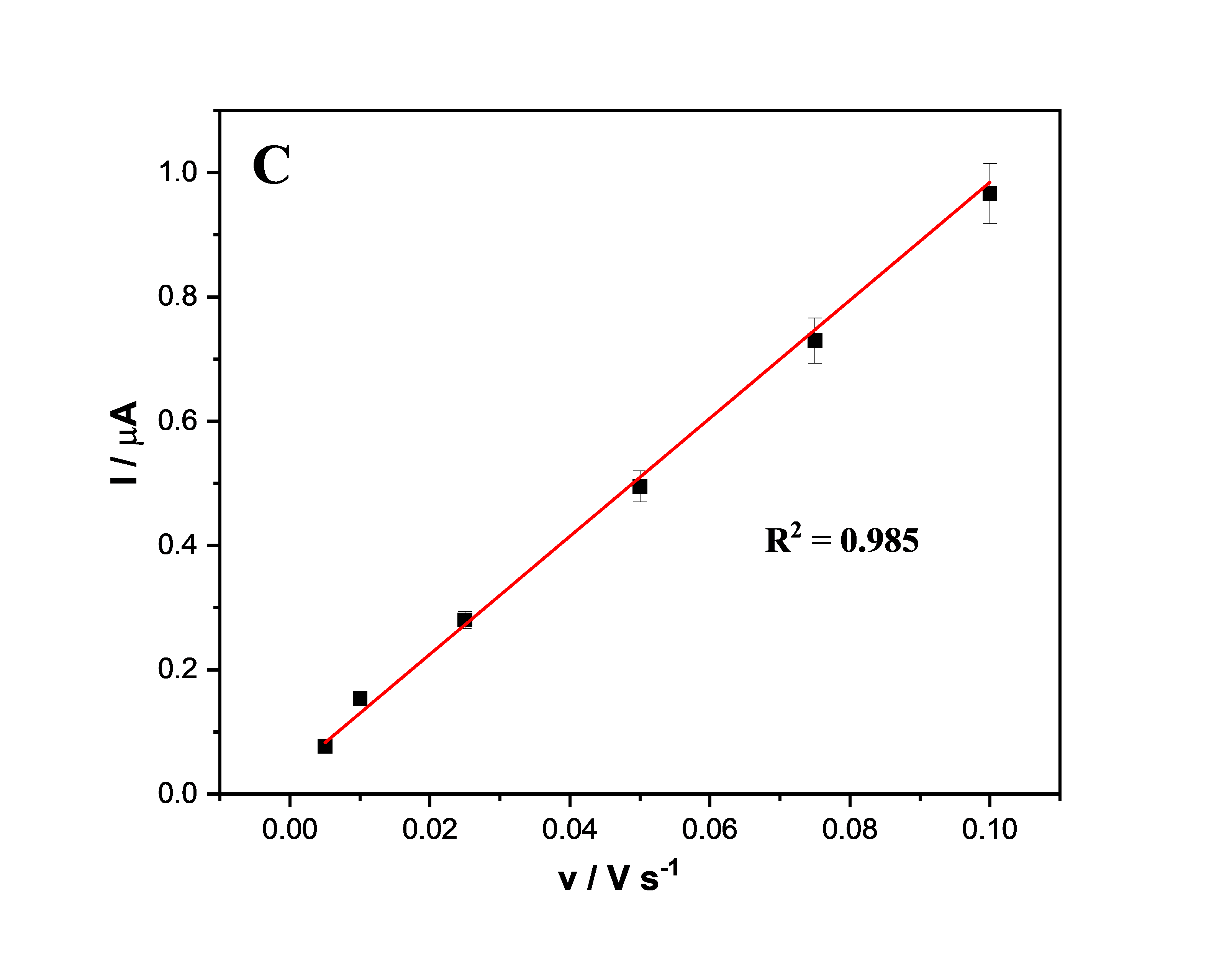


**Fig. S6 –** (A) Cyclic voltammetric responses recorded for 10.0 µmol L⁻¹ MFA in 0.1 mol L⁻¹ PBS (pH 7) at scan rates from 5 to 100 mV s⁻¹, using a step potential of 2 mV upon SPCE-Gr/NiNPs. (B) Plot of log I (µA) versus log v (V s⁻¹). (C) Plot of I (µA) versus v (V s⁻¹). Data corresponds to the mean values of triplicate measurements (n = 3); error bars represent the standard deviation.


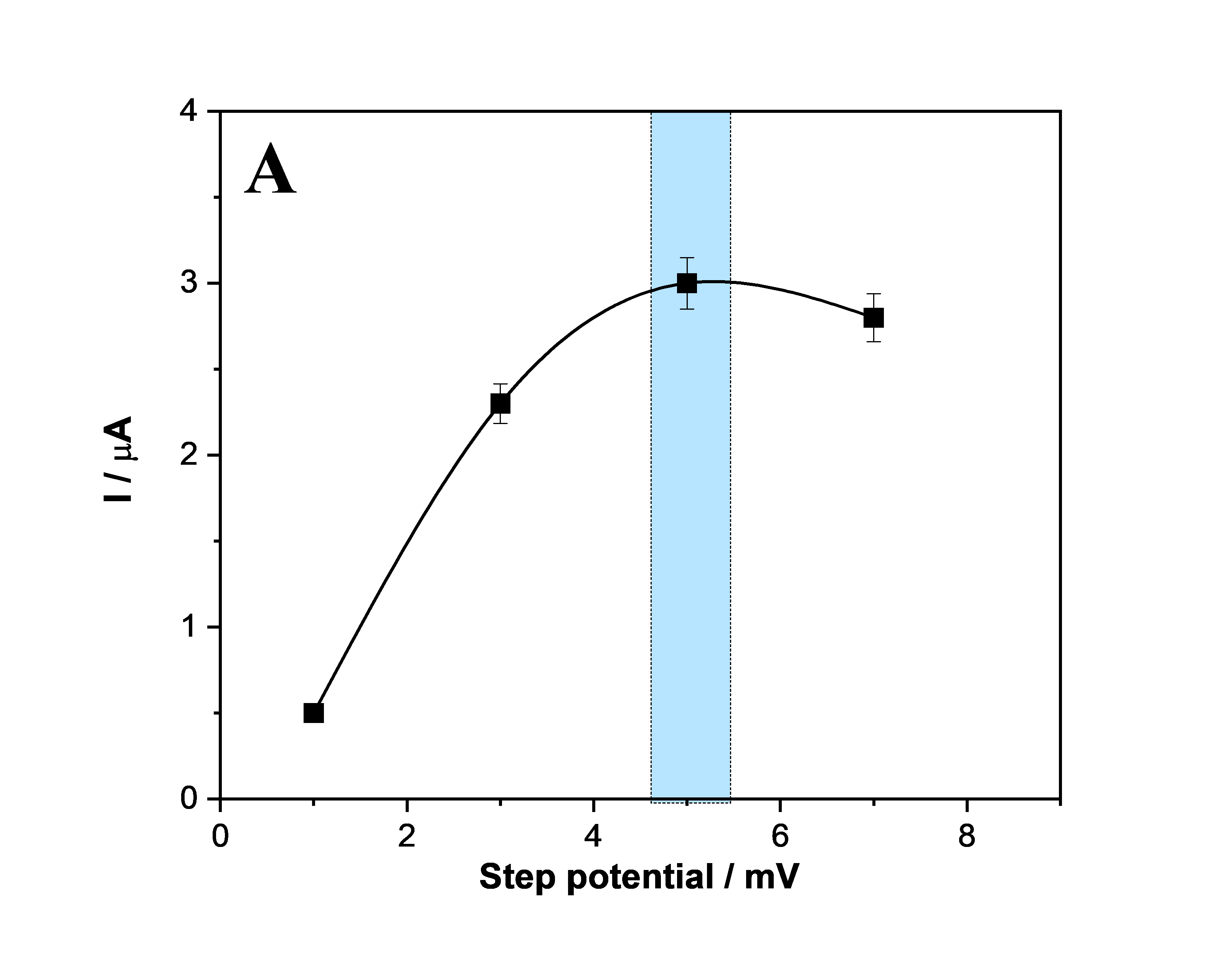

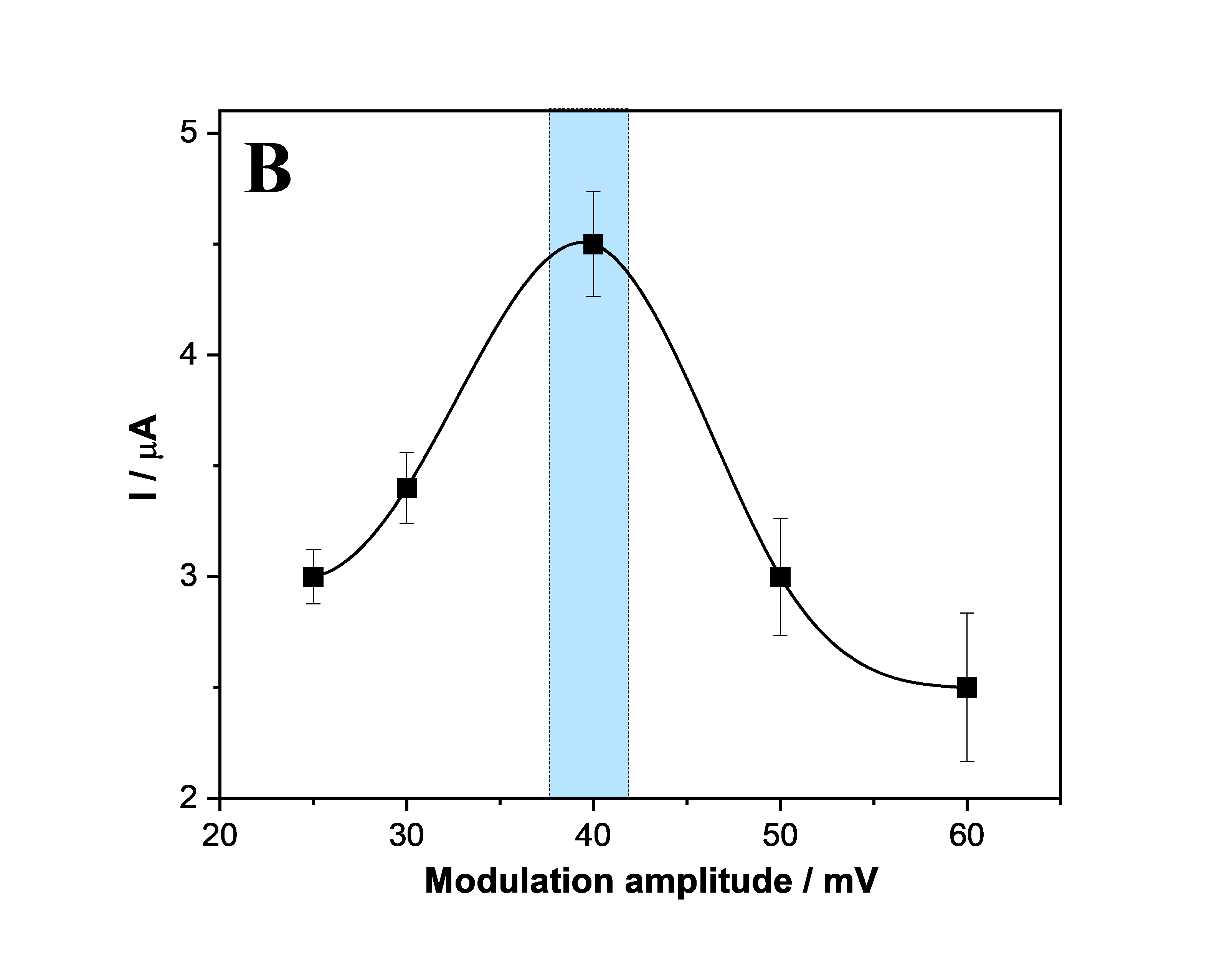

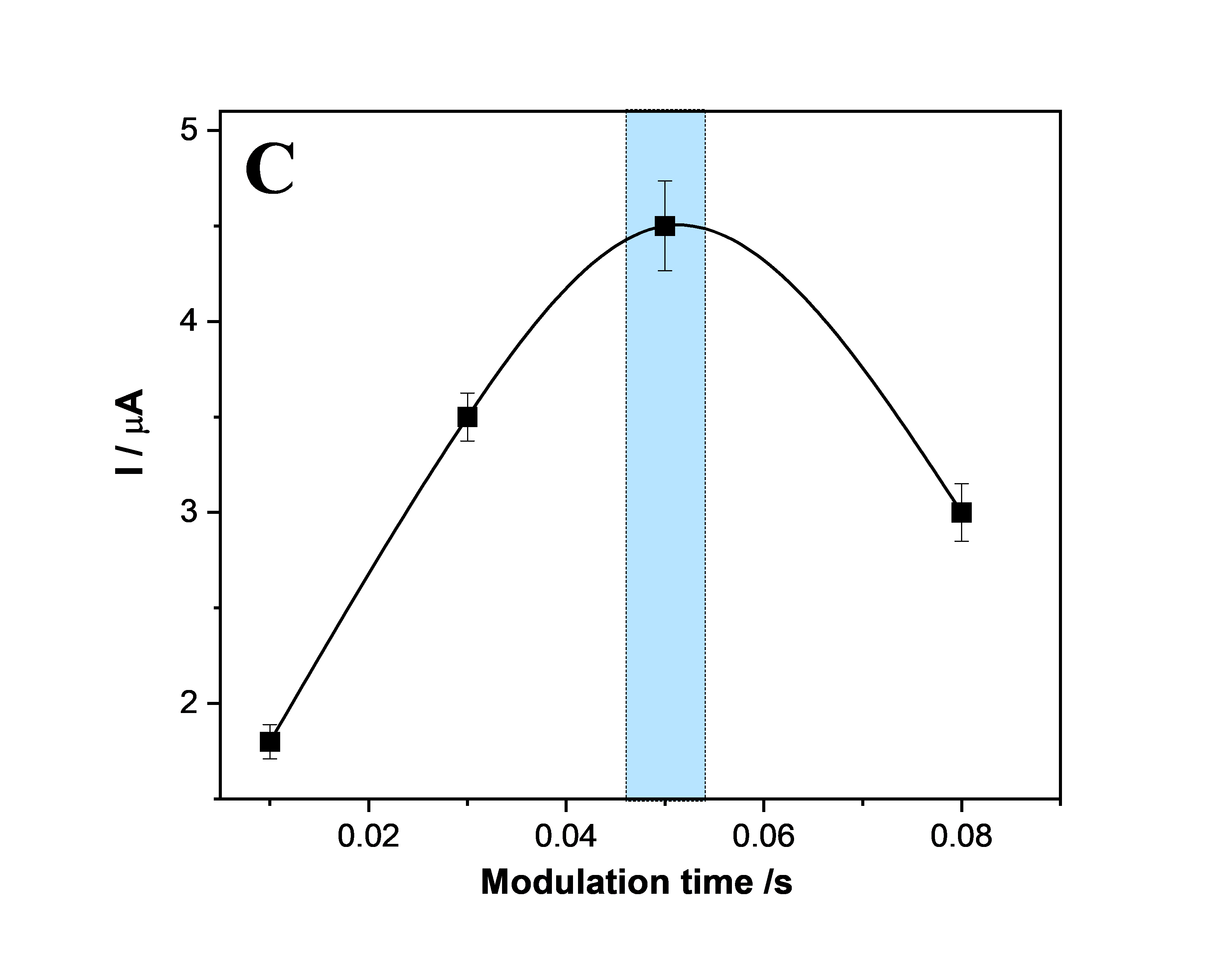


**Fig. S7 –** Impact of the DPV parameters (A) step potential (1 to 7 mV), (B) modulation amplitude (25 to 60 mV), (C) modulation time (0.01 to 0.08 s) on the electrochemical response of 10.0 µmol L⁻¹ MFA in 0.1 mol L⁻¹ PBS (pH 7). Data corresponds to the mean values of triplicate measurements (n = 3); error bars represent the standard deviation.


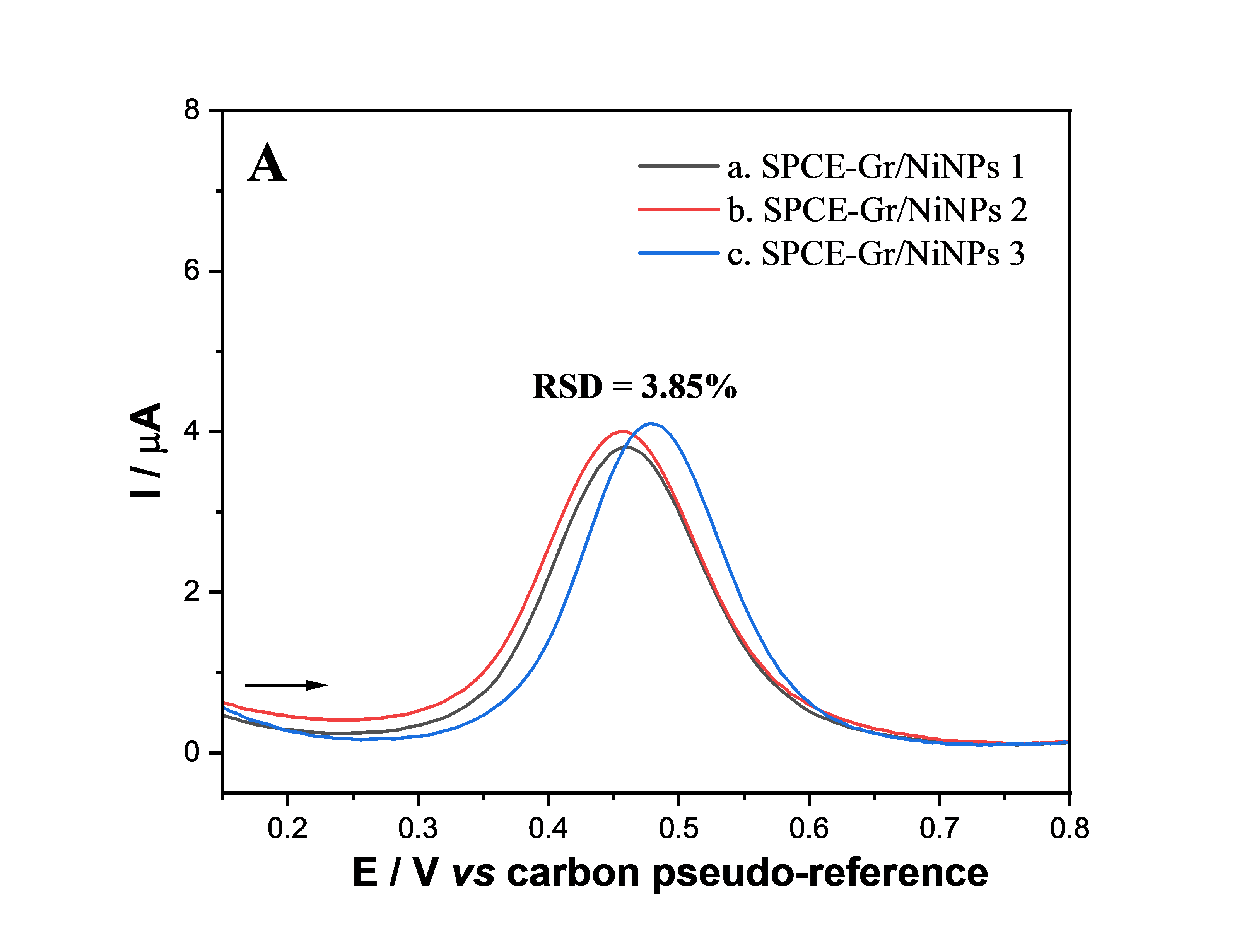

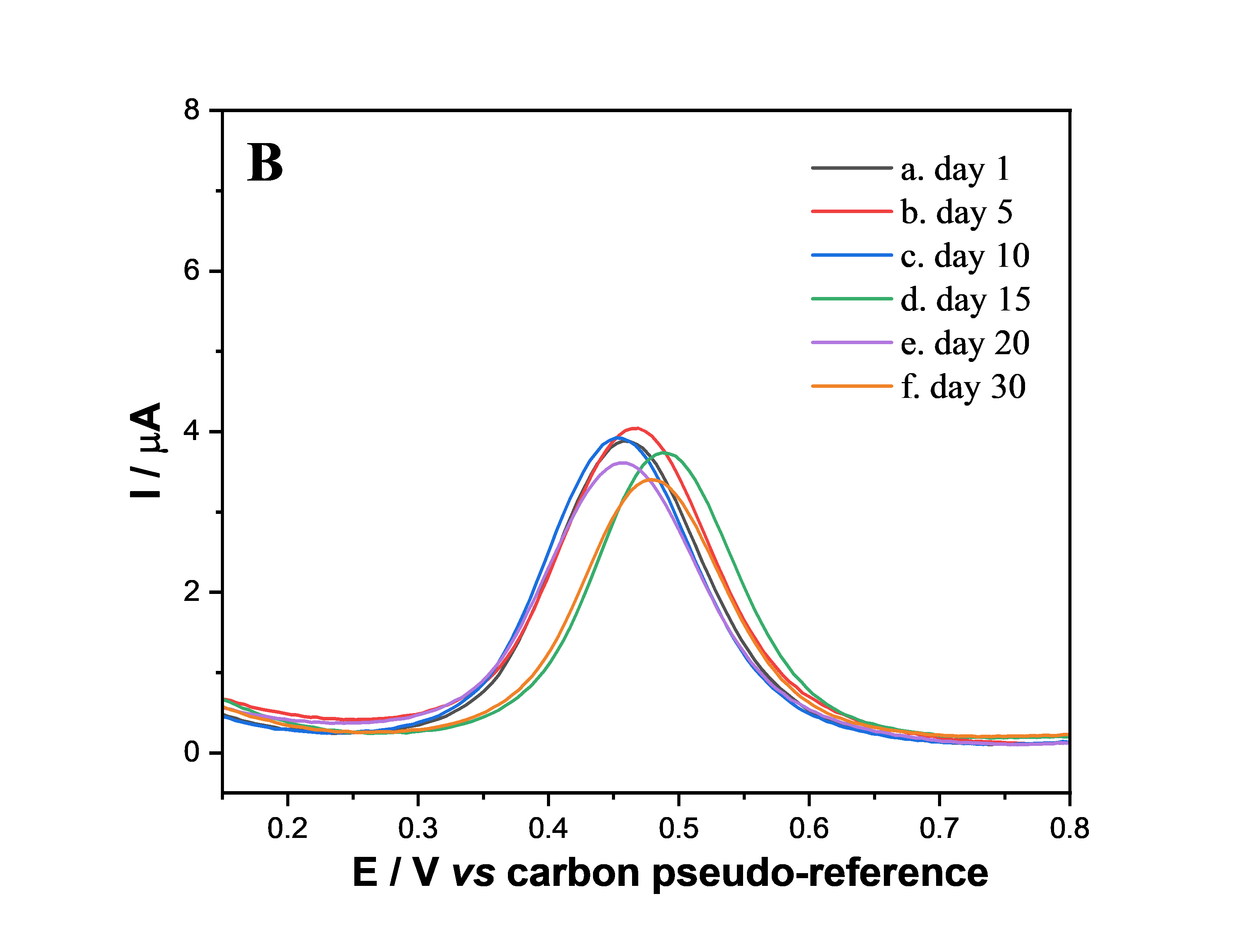

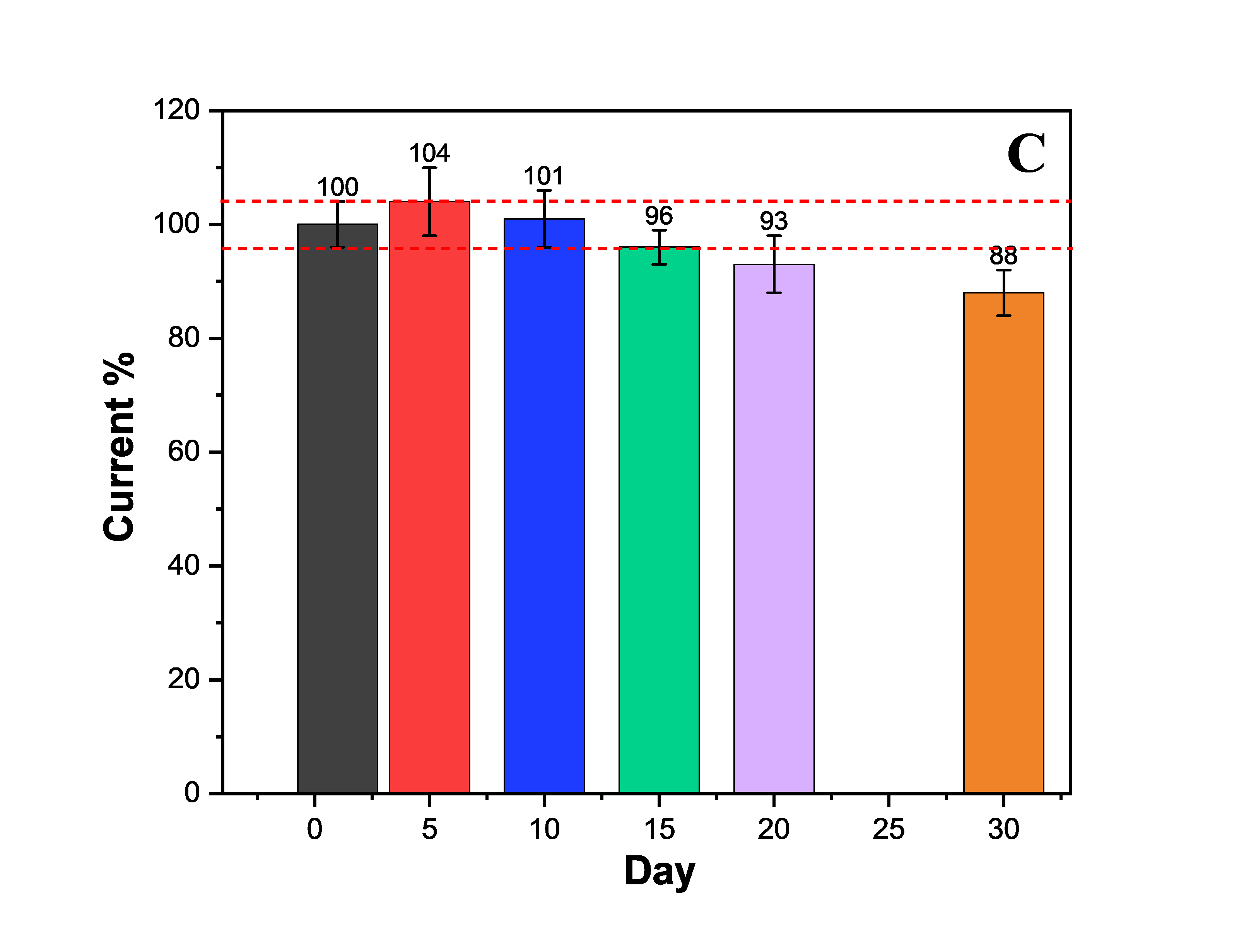


**Fig. S8 –** DPV data of the SPCE-Gr/NiNPs device recorded in the presence of 9.0 µmol L⁻¹ MFA in 0.1 mol L⁻¹ PBS at pH 7: (A) Reproducibility study evaluated through consecutive DPV measurements using three independently prepared SPCE-Gr/NiNPs electrodes (n = 3) under identical experimental conditions. (B–C) Stability study of the sensor response over time, based on DPV measurements recorded from day 1 to day 30 (n = 3, intra-day), illustrating the variation in the electrochemical signal throughout the evaluation period.


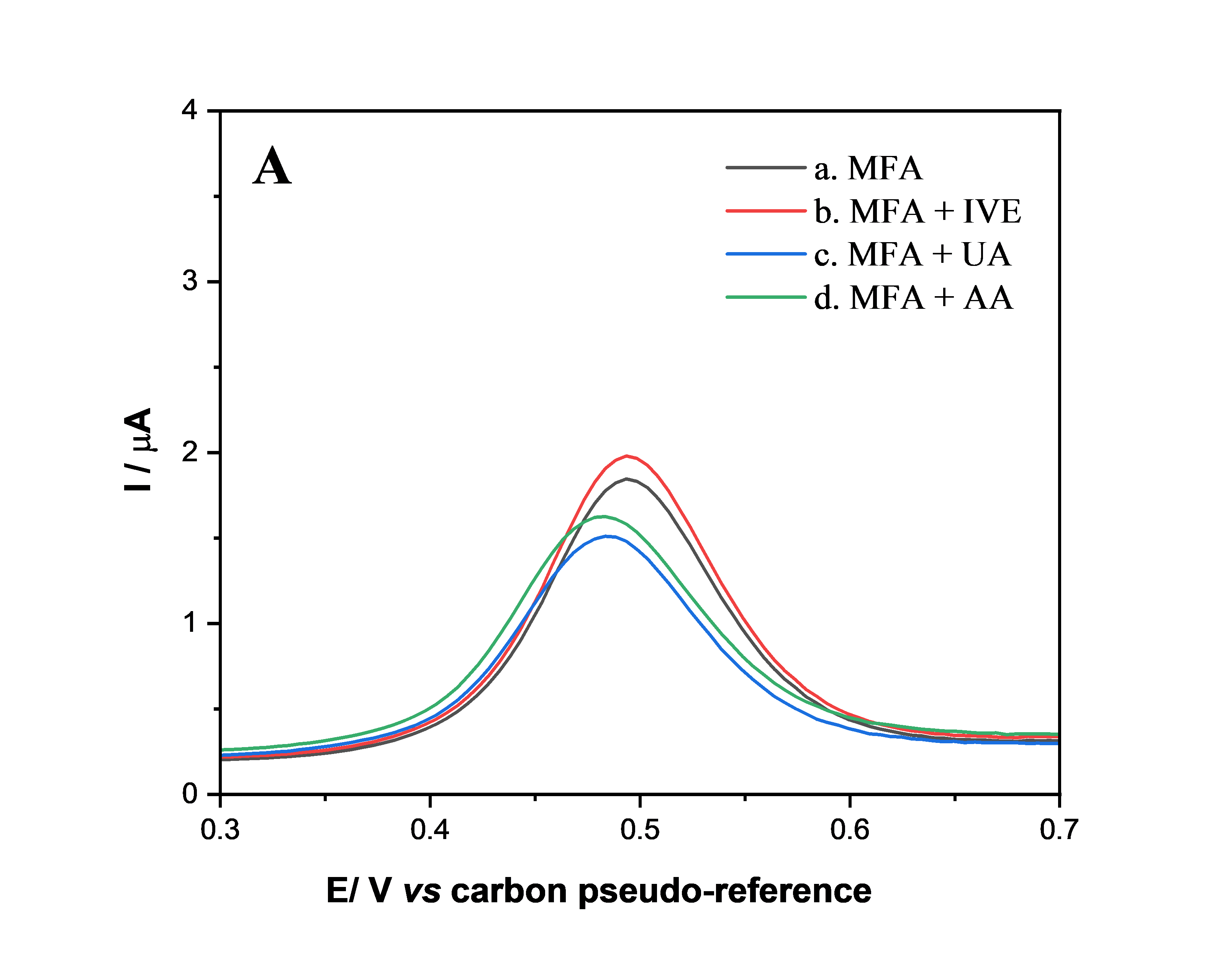

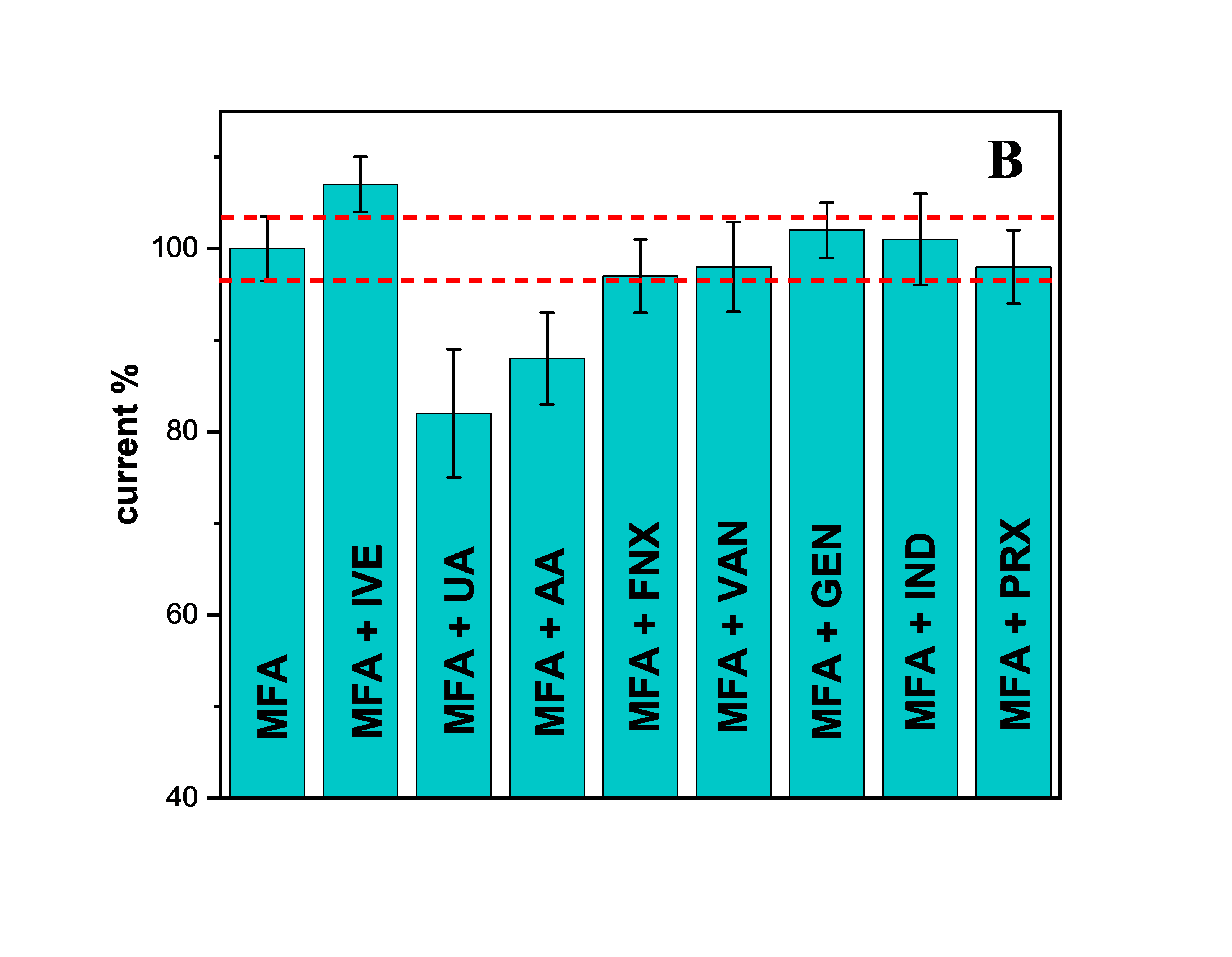


**Fig. S9 -** Selectivity analysis of 4.0 µmol L⁻¹ MFA in 0.1 mol L⁻¹ PBS (pH 7) in the presence of possible interferents at 10-fold higher concentrations: (A) DPV responses of MFA in the presence of some interfering compounds (IVE, UA, and AA). (B) Bar graph of the percentage current response for MFA alone and MFA in the presence of each interferent agent.


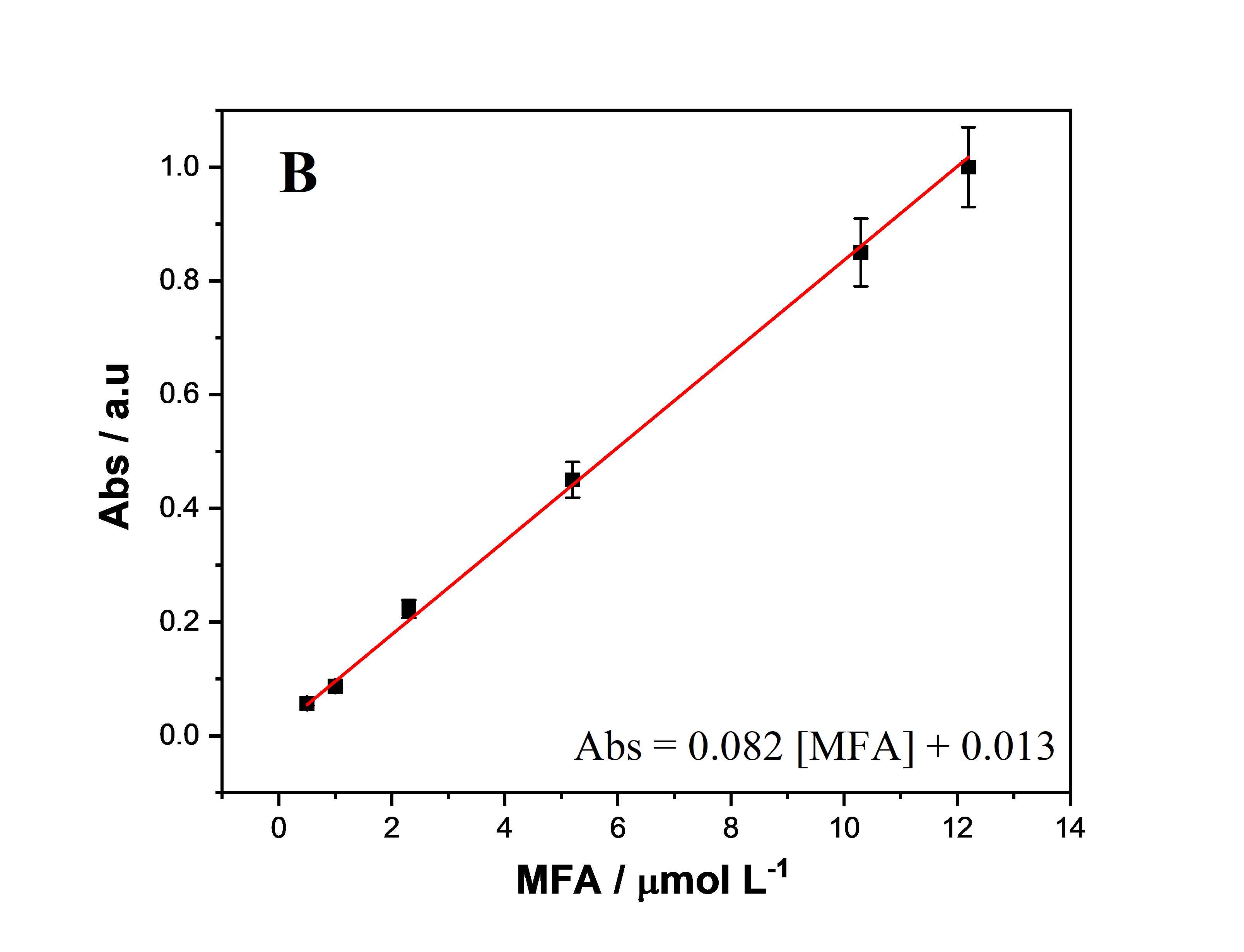

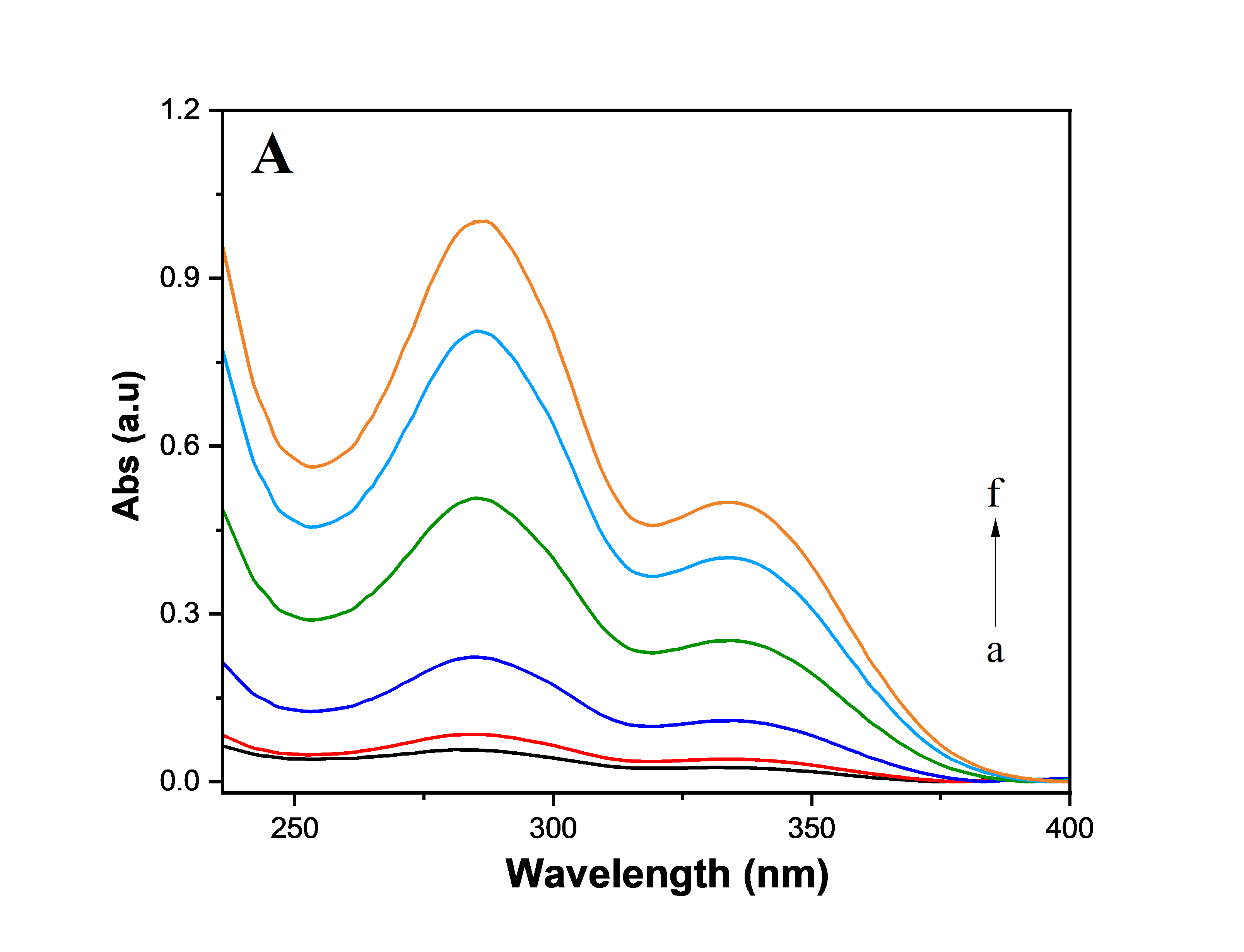


**Figure S10 –** (A) UV-Vis absorption spectra of the MFA at a 235 to 400 nm using concentration levels of MFA between 0.5 and 12.20 µmol L^-1^ (a-f); (B) Respective calibration curve obtained.
